# Sulfur filling activates vacancy-induced C–C bond cleavage in polyol electrooxidation

**DOI:** 10.1093/nsr/nwae271

**Published:** 2024-08-05

**Authors:** Jianqiao Shi, Wei Chen, Yandong Wu, Yanwei Zhu, Chao Xie, Yimin Jiang, Yu-Cheng Huang, Chung-Li Dong, Yuqin Zou

**Affiliations:** State Key Laboratory of Chemo/Bio-Sensing and Chemometrics, College of Chemistry and Chemical Engineering, Advanced Catalytic Engineering Research Center of the Ministry of Education, Hunan University, Changsha 410082, China; State Key Laboratory of Chemo/Bio-Sensing and Chemometrics, College of Chemistry and Chemical Engineering, Advanced Catalytic Engineering Research Center of the Ministry of Education, Hunan University, Changsha 410082, China; State Key Laboratory of Chemo/Bio-Sensing and Chemometrics, College of Chemistry and Chemical Engineering, Advanced Catalytic Engineering Research Center of the Ministry of Education, Hunan University, Changsha 410082, China; State Key Laboratory of Chemo/Bio-Sensing and Chemometrics, College of Chemistry and Chemical Engineering, Advanced Catalytic Engineering Research Center of the Ministry of Education, Hunan University, Changsha 410082, China; State Key Laboratory of Chemo/Bio-Sensing and Chemometrics, College of Chemistry and Chemical Engineering, Advanced Catalytic Engineering Research Center of the Ministry of Education, Hunan University, Changsha 410082, China; State Key Laboratory of Chemo/Bio-Sensing and Chemometrics, College of Chemistry and Chemical Engineering, Advanced Catalytic Engineering Research Center of the Ministry of Education, Hunan University, Changsha 410082, China; Research Center for X-ray Science & Department of Physics, Tamkang University, New Taipei City 25137, China; Research Center for X-ray Science & Department of Physics, Tamkang University, New Taipei City 25137, China; State Key Laboratory of Chemo/Bio-Sensing and Chemometrics, College of Chemistry and Chemical Engineering, Advanced Catalytic Engineering Research Center of the Ministry of Education, Hunan University, Changsha 410082, China

**Keywords:** oxygen vacancy, vacancy filling, alcohol electrooxidation, polyol electrooxidation, C–C bond cleavage

## Abstract

Using the electrochemical polyol oxidation reaction (POR) to produce formic acid over nickel-based oxides/hydroxides (NiO*_x_*H*_y_*) is an attractive strategy for the electrochemical upgrading of biomass-derived polyols. The key step in the POR, i.e. the cleavage of the C–C bond, depends on an oxygen-vacancy-induced mechanism. However, a high-energy oxygen vacancy is usually ineffective for Schottky-type oxygen-vacancy-rich β-Ni(OH)_2_ (V_SO_-β-Ni(OH)_2_). As a result, both β-Ni(OH)_2_ and V_SO_-β-Ni(OH)_2_ cannot continuously catalyze oxygen-vacancy-induced C–C bond cleavage during PORs. Here, we report a strategy of oxygen-vacancy-filling with sulfur to synthesize a β-Ni(OH)_2_ (S-V_O_-β-Ni(OH)_2_) catalyst, whose oxygen vacancies are protected by filling with sulfur atoms. During PORs over S-V_O_-β-Ni(OH)_2_, the pre-electrooxidation-induced loss of sulfur and structural self-reconstruction cause the *in-situ* generation of stable Frenkel-type oxygen vacancies for activating vacancy-induced C–C bond cleavage, thus leading to excellent POR performances. This work provides an intelligent approach for guaranteeing the sustaining action of the oxygen-vacancy-induced catalytic mechanism in electrooxidation reactions.

## Teaser

Pre-electrooxidation-induced loss of filling sulfur atoms in S-VO-β-Ni(OH)_2_ generates stable Frenkel-type oxygen vacancies for activating vacancy-induced C–C bond cleavage, which eliminates the passivation of β-Ni(OH)_2_ during polyol electrooxidation reaction.

## INTRODUCTION

With the development of defect chemistry, researchers have recognized the important role of oxygen vacancies (V_O_) in electrocatalysis based on defective nano-catalysts [1–3]. Simplistically, oxygen vacancies can be divided into two forms: (i) Schottky-type oxygen vacancies are formed via the escape of lattice oxygen atoms; (ii) Frenkel-type oxygen vacancies are generated due to the transformation of lattice oxygen to interstitial oxygen [4,5]. The fabrication of oxygen-vacancy-rich catalysts (V_O_-catalysts) is a crucial modification strategy for enhancing catalytic performance and regulating the reaction pathway [6,7]. However, high-energy oxygen vacancies easily lose efficacy, leading to the gradual decline in activity of V_O_-catalysts, especially for those catalytic reactions involving V_O_-induced mechanisms [8,9]. As to the development of V_O_-catalysts, the chief difficulty is how to ensure the sustaining action of the oxygen vacancy during catalytic reactions.

As a typical structure-sensitive reaction, the electrochemical alcohol oxidation reaction (AOR) on nickel-based catalysts provides an important method for oxidative upgrading of biomass-derived alcohols, e.g. primary alcohol and polyols [3,10,11]. Reaction pathways of AORs over nickel-based catalysts change as alcohol substrates change [12,13]. The electrochemical primary alcohol oxidation reaction (PAOR) is a dehydrogenation reaction, and the product of the PAOR is carboxylic acid (R–COOH) [14,15]. The electrochemical polyol oxidation reaction (POR) involves the C–C bond cleavage, and the electrooxidation product of polyols is formic acid (FA; HCOOH) [13,16]. Our previous work has demonstrated that the oxygen-vacancy-induced catalytic mechanism plays a critical role in the oxidative cleavage of the C–C bond during PORs on NiO*_x_*H*_y_* species [3]. Hence, stable V_O_ is necessary for PORs on nickel-based catalysts involving the C–C bond cleavage.

Although V_O_-catalysts are easy to manufacture, an unstable oxygen vacancy (especially for Schottky-type defects) is easily filled with reactive oxygen species (e.g. oxygen) upon exposure to atmospheres, thus leading to V_O_-catalyst deactivation [9]. The key to developing PORs over nickel-based catalysts is to focus on how to stabilize oxygen vacancies to guarantee the sustaining C–C bond cleavage during PORs. It is hard to reconcile the stability of the oxygen vacancy with its high activity; hence, the direct preparation of stable oxygen vacancies is a challenging task. Stocking oxygen vacancies with removable heteroatoms may be an intelligent strategy for developing highly efficient POR catalysts [17]. In theory, stocking Schottky-type oxygen vacancies in NiO*_x_*H*_y_* with heteroatoms not only protects unstable oxygen vacancies but also guarantees the *in-situ* preparation of oxygen vacancies during the POR.

Here, we synthesize Schottky-type oxygen-vacancy-rich β-Ni(OH)_2_ (V_SO_-β-Ni(OH)_2_) via low-temperature plasma-etching, and fill oxygen vacancies in V_SO_-β-Ni(OH)_2_ with removable sulfur (S) heteroatoms to synthesize oxygen-vacancy-filled β-Ni(OH)_2_ (S-V_O_-β-Ni(OH)_2_). *Ex/in-situ* characterizations proved that Schottky-type oxygen vacancies in V_SO_-β-Ni(OH)_2_ were easily deactivated, thus leading to the electrode passivation during PORs over V_SO_-β-Ni(OH)_2_. As to PORs over S-V_O_-β-Ni(OH)_2_, the electrooxidation-induced dissolution of filling S atoms causes the *in-situ* generation of stable Frenkel-type oxygen vacancies (V_FO_), which can continuously catalyze the C–C bond cleavage. Hence, S-V_O_-β-Ni(OH)_2_ can efficiently catalyze the electrooxidation of polyols to FA, thus exhibiting excellent POR performance without the electrode passivation. By combining the experimental and calculation results, this work illustrates the detailed reaction pathway of PORs over S-V_O_-β-Ni(OH)_2_, which provides important theoretical support for the general design strategy of POR catalysts. This intelligent method of filling oxygen vacancies with heteroatoms not only protects unstable oxygen vacancies in catalysts, but also guarantees the sustained effect of the oxygen-vacancy-induced catalytic mechanism in electrooxidation reactions.

## RESULTS AND DISCUSSION

### Fabrication and characterization of S-V_O_-β-Ni(OH)_2_

**Figure 1.**
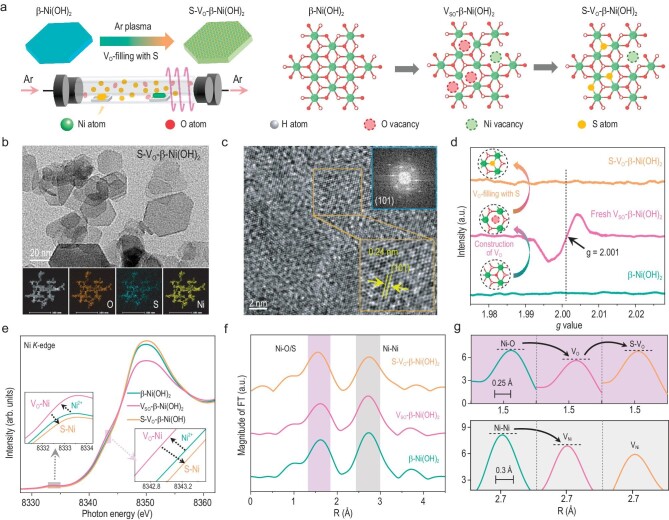
Synthesis and characterizations of the catalysts. (a) Schematic illustration of the plasma-assisted synthesis of V_SO_-β-Ni(OH)_2_ and S-V_O_-β-Ni(OH)_2_. (b) Transmission electron microscopy (TEM) and EDX images of S-V_O_-β-Ni(OH)_2_ nanosheets. (c) High-resolution TEM (HR-TEM) images and selected-area electron diffraction (SAED) pattern of S-V_O_-β-Ni(OH)_2_ nanosheets. (d–f) EPR (d), Ni K-edge XANES spectra (e), and the magnitude of the Fourier transform for Ni K-edge EXAFS spectra (f) of β-Ni(OH)_2_, V_SO_-β-Ni(OH)_2_ and S-V_O_-β-Ni(OH)_2_. (g) Magnified views of the boxed areas in Fig. 1f.

Plasma treatment is a promising method for the fabrication of oxygen vacancies, primarily creating Schottky-type oxygen vacancies [18,19]. In theory, V_SO_-β-Ni(OH)_2_ can be synthesized via etching β-Ni(OH)_2_ with argon (Ar) plasma. However, an unstable Schottky-type oxygen vacancy (V_SO_) is easily filled with reactive oxygen species upon exposure to atmospheres, leading to deactivation of the catalyst. Using a thermally coupled Ar plasma modification approach with sublimation sulfur (S) as a sulfur source, excited S atoms were filled into generated V_SO_, thus obtaining oxygen-vacancy-filling with sulfur β-Ni(OH)_2_ (S-V_O_-β-Ni(OH)_2_) (Fig. 1a) [20]. By using multiple material characterization techniques, we found that the crystal structure and morphology of β-Ni(OH)_2_, V_SO_-β-Ni(OH)_2_ and S-V_O_-β-Ni(OH)_2_ nanosheets were nearly identical, indicating that the microstructure of samples remained basically unchanged during the fabrication of S-V_O_-β-Ni(OH)_2_ (Fig. 1b and c, and Figs S1–S3). X-ray photoelectron spectroscopy (XPS) spectra and energy-dispersive X-ray (EDX) spectrum mapping confirmed that S atoms were, indeed, introduced into the S-V_O_-β-Ni(OH)_2_ sample, and S atoms were evenly distributed on S-V_O_-β-Ni(OH)_2_ nanosheets (Figs S4 and S5, and Tables S1 and S2).

Electron paramagnetic resonance (EPR) spectroscopy was carried out to identify oxygen vacancies [21]. The symmetrical EPR signal at *g* = ∼2.001 is attributed to the oxygen vacancies because unpaired electrons are trapped at oxygen vacancies [22]. According to EPR spectra, the fresh V_SO_-β-Ni(OH)_2_ sample contains abundant oxygen vacancies while there are few oxygen vacancies for β-Ni(OH)_2_ and S-V_O_-β-Ni(OH)_2_, indicating that, during the fabrication of S-V_O_-β-Ni(OH)_2_, active oxygen vacancies in V_SO_-β-Ni(OH)_2_ have been filled with S atoms (Fig. 1d). X-ray absorption near edge structure (XANES) measurements were performed to further confirm the local atomic and electronic structures of catalysts (Fig. S6) [23]. Compared with β-Ni(OH)_2_, both the absorption edge and pre-edge peak in the Ni K-edge XANES spectra of V_SO_-β-Ni(OH)_2_ are shifted to lower energies because V_SO_ causes coordination-unsaturated Ni cations with lower valence states (Fig. 1e) [24]. According to the Ni K-edge XANES spectra of S-V_O_-β-Ni(OH)_2_, both absorption edge and pre-edge peak are shifted back to high energies, indicating that V_SO_ in S-V_O_-β-Ni(OH)_2_ has been filled with S heteroatoms (Fig. 1e) [20].

Extended X-ray absorption fine structure (EXAFS) spectroscopy provides detailed information on the coordination structures of catalysts [25]. There are two dominant peaks in the magnitude of the Fourier transform for Ni K-edge EXAFS spectra of β-Ni(OH)_2_, V_SO_-β-Ni(OH)_2_ and S-V_O_-β-Ni(OH)_2_ (Fig. S7 and Table S3). The first and second peaks are attributed to the first shell contribution (e.g. Ni–O and Ni–S) and the second Ni–Ni shell contribution (e.g. Ni–O–Ni and Ni–S–Ni), respectively [26]. The intensities of the two peaks of V_SO_-β-Ni(OH)_2_ are significantly lower than those of β-Ni(OH)_2_, indicating that the average coordination numbers of Ni–O and Ni–Ni in V_SO_-β-Ni(OH)_2_ are significantly lower than those in β-Ni(OH)_2_ (Fig. 1f and Fig. S7) [27]. Oxygen and nickel vacancies cause a decrease in coordination numbers of Ni–O and Ni–Ni, respectively; hence, EXAFS results fully prove that the V_SO_-β-Ni(OH)_2_ sample is rich in nickel vacancies and Schottky-type oxygen vacancies [28]. Compared with V_SO_-β-Ni(OH)_2_, the intensity of the first shell contribution of S-V_O_-β-Ni(OH)_2_ is greatly increased while the intensity of the second Ni–Ni shell contribution remains relatively constant (Fig. 1g). This directly proves that, for S-V_O_-β-Ni(OH)_2_, S atoms are filled into Schottky-type oxygen vacancies, instead of nickel vacancies, to generate the first shell Ni–S bonds.

### Identifying electrooxidation-induced oxygen vacancies

**Figure 2.**
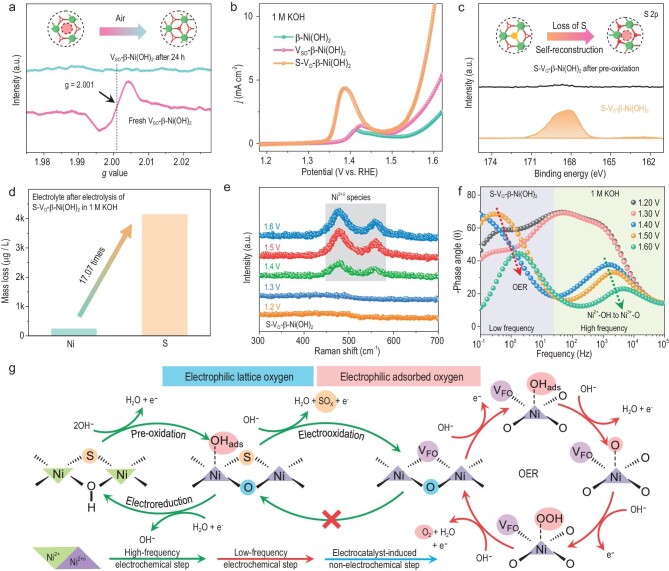
*In-situ* generation of oxygen vacancies during electrooxidation over S-V_O_-β-Ni(OH)_2_. (a) EPR spectra of the fresh V_SO_-β-Ni(OH)_2_ sample and the V_SO_-β-Ni(OH)_2_ sample after being exposed to air for 24 hours. (b) Anodic polarization curves of β-Ni(OH)_2_, V_SO_-β-Ni(OH)_2_ and S-V_O_-β-Ni(OH)_2_ in 1 M KOH. (c) XPS spectra of S 2p for S-V_O_-β-Ni(OH)_2_ and the S-V_O_-β-Ni(OH)_2_ sample after pre-oxidation at 1.40 V vs. reversible hydrogen electrode (RHE) in 1 M KOH. (d) Inductively coupled plasma mass spectrometry (ICP-MS) spectra for the electrolyte after electrolysis using S-V_O_-β-Ni(OH)_2_ (1.40 V vs. RHE). (e) *In-situ* Raman spectra of S-V_O_-β-Ni(OH)_2_ in 1 M KOH with different potentials. (f) Bode plots of the OER system based on S-V_O_-β-Ni(OH)_2_ at different potentials. (g) Schematic diagram of surface species evolution in the OER system based on S-V_O_-β-Ni(OH)_2_.

Although the fresh V_SO_-β-Ni(OH)_2_ sample initially contains a few Schottky-type oxygen vacancies, almost all V_SO_ have been eliminated by reactive oxygen species when the V_SO_-β-Ni(OH)_2_ sample was exposed to air for 24 hours (Fig. 2a). Because the Ni^3+^ species is the key reaction intermediate for AORs over NiO*_x_*H*_y_*, the Ni^2+^/Ni^3+^ redox couple is considered an important reference for estimating the AOR performance of nickel-based catalysts [25]. According to anodic polarization curves of the catalysts in 1 M KOH, the Ni^2+^/Ni^3+^ redox potential of S-V_O_-β-Ni(OH)_2_ is significantly lower than that of β-Ni(OH)_2_ and V_SO_-β-Ni(OH)_2_, and the performance of the oxygen evolution reaction (OER) for S-V_O_-β-Ni(OH)_2_ is optimal among three catalysts (Fig. 2b, Figs S8 and S9).

Because of the Ni^2+^/Ni^3+^ redox, electrooxidation reactions on S-V_O_-β-Ni(OH)_2_ usually involve a pre-electrooxidation reaction, i.e. the electrooxidation of the catalyst to generate Ni^2+δ^O_*x*_H_*y*_ species (Fig. S10) [29,30]. Furthermore, we traced the evolution of S atoms during the pre-electrooxidation of S-V_O_-β-Ni(OH)_2_ in 1 M KOH. According to EDX images and XPS spectra, there are few S atoms on the surface of the S-V_O_-β-Ni(OH)_2_ electrode after pre-electrooxidation (Fig. 2c, Fig. S11 and Table S4). ICP-MS detected large amounts of dissolved S atoms in the electrolyte after pre-electrooxidation, corroborating the results of XPS and EDX measurements (Fig. 2d). These results prove that S atoms filled in oxygen vacancies can be electrochemically oxidized to generate dissoluble sulfur oxides (SO*_x_*) during pre-electrooxidation of S-V_O_-β-Ni(OH)_2_ so that sulfur-loss-induced oxygen vacancies (S-V_O_) can be exposed again [31]. Therefore, S atoms filling in S-V_O_-β-Ni(OH)_2_ offer protection for unstable oxygen vacancies, and protected oxygen vacancies in S-V_O_-β-Ni(OH)_2_ can be exposed *in situ* during electrooxidation.

S-V_O_ formation depends on the electrooxidation of S-V_O_-β-Ni(OH)_2_, i.e. the electrooxidation of Ni^2+^ species to Ni^3+^ species [32,33]. The generation of Schottky-type oxygen vacancies is often accompanied by a decrease in the Ni valence state [34,35]. Hence, the structure of S-V_O_ in S-V_O_-β-Ni(OH)_2_ after sulfur leaching differs from that of unstable Schottky-type oxygen vacancies in V_SO_-β-Ni(OH)_2_. The formation of high-valence Ni species indicates that, during the electrooxidation-induced structural self-reconstruction of S-V_O_-β-Ni(OH)_2_, some oxygenated species (e.g. OH_ads_) were transformed into interstitial oxygen atoms to generate Frenkel-type oxygen vacancies (V_FO_) instead of Schottky-type oxygen vacancies [36]. Due to the existence of interstitial oxygen atoms, it is difficult for Frenkel-type S-V_O_ to be oxidized by oxygen-containing species (e.g. oxygen). Consequently, S-V_O_ in S-V_O_-β-Ni(OH)_2_, after sulfur leaching, is relatively stable under ambient atmosphere, which is radically different from oxygen vacancies in V_SO_-β-Ni(OH)_2_. The stability of S-V_O_ is one of the reasons why S-V_O_-β-Ni(OH)_2_ exhibits excellent electrochemical performance in PORs without the electrode passivation.

*In-situ* Raman spectra show that high-valance nickel (Ni^2+δ^) species containing electrophilic oxygen can be generated and accumulated at a potential above 1.35 V in the OER system based on S-V_O_-β-Ni(OH)_2_ (Fig. 2e). However, *in-situ* Raman spectra cannot distinguish between electrophilic adsorbed and lattice oxygen species (e.g. Ni^3+^–O bonds and Ni^2+δ^–OH_ads_). Operando electrochemical impedance spectroscopy (EIS) is an effective tool for identifying electrochemical and interfacial behaviors during electrochemical reactions [30,37]. As to the OER system based on S-V_O_-β-Ni(OH)_2_, EIS identified two different electron transfer processes, including (i) a high-frequency electrochemical step occurring at a potential above 1.35 V, and (ii) a low-frequency electrochemical step occurring at a potential above 1.5 V (Fig. 2f and Fig. S12). In view of their potential range, the electrochemical generation of electrophilic lattice oxygen species (i.e. Ni^3+^–O) and the OER involving electrophilic adsorbed oxygen species (e.g. Ni^2+δ^–OH_ads_) are considered high-frequency and low-frequency electrochemical steps, respectively (Fig. S12). Hence, based on frequency-dependent impedance responses, we can effectively distinguish between electrophilic lattice oxygen species and electrophilic adsorbed oxygen species during electrooxidation reactions on S-V_O_-β-Ni(OH)_2_. To sum up, the OER system based on S-V_O_-β-Ni(OH)_2_ includes two processes: (i) the pre-electrooxidation reaction, including the *in-situ* generation of S-V_O_ and the electrochemical dehydrogenation of the lattice hydroxy group (Ni^2+^–OH) to a Ni^3+^–O bond containing electrophilic lattice oxygen; (ii) the OER involving electrophilic adsorbed oxygen intermediates (e.g. Ni^2+δ^–OH_ads_) occurring at the low-frequency interface (Fig. 2g).

### Catalytic role of S-V_O_ in the PAOR

**Figure 3.**
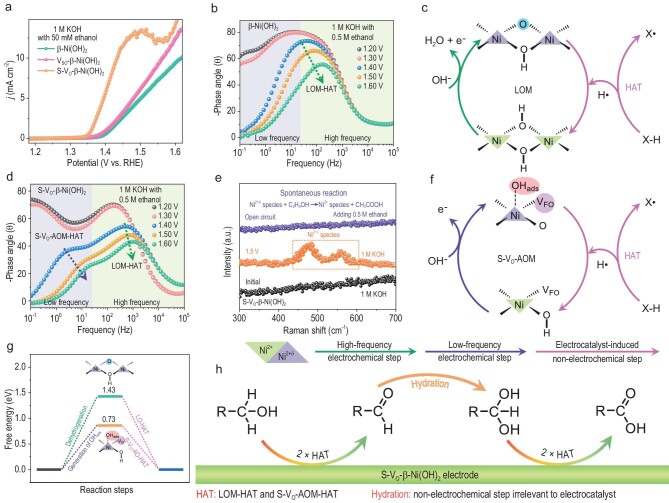
Catalyst functions of β-Ni(OH)_2_ and S-V_O_-β-Ni(OH)_2_ in the PAOR. (a) Anodic polarization curves of β-Ni(OH)_2_, V_SO_-β-Ni(OH)_2_ and S-V_O_-β-Ni(OH)_2_ in the model PAOR system (1 M KOH with 50 mM ethanol). (b) Bode plots of the PAOR over β-Ni(OH)_2_ at different potentials (1 M KOH with 0.5 M ethanol). (c) Schematic diagram of LOM-HAT, comprising the electrochemical generation of Ni^3+^–O and LO-HAT. (d) Bode plots of the PAOR over S-V_O_-β-Ni(OH)_2_ at different potentials. (e) *In-situ* Raman spectra of S-V_O_-β-Ni(OH)_2_ in the electrochemical testing, which is shown in Fig. S24a. (f) Schematic diagram of S-V_O_-AOM-HAT, comprising the electrochemical generation of S-V_O_-Ni^2+δ^-OH_ads_ and S-V_O_-AO-HAT. (g) DFT calculations for the dehydrogenation of Ni^2+^–OH to Ni^3+^–O over β-Ni(OH)_2_ and the generation of S-V_O_-Ni^2+δ^-OH_ads_ over S-V_O_-β-Ni(OH)_2_. (h) Diagram illustrating the reaction pathway of PAORs on S-V_O_-β-Ni(OH)_2_.

Ethanol was used as a model primary alcohol (R–CH_2_OH) substrate to explore the reaction mechanism of the PAOR. Due to the inactivation of high-energy V_SO_, the intrinsic activity of the PAOR over V_SO_-β-Ni(OH)_2_ is similar to that on β-Ni(OH)_2_ (Fig. 3a and Fig. S13). The intrinsic activity of the PAOR over S-V_O_-β-Ni(OH)_2_ far outweighs that over V_SO_-β-Ni(OH)_2_ or β-Ni(OH)_2_, and the onset potential of the PAOR is reduced from 1.40 V to 1.35 V, indicating that *in-situ*-generated V_FO_ greatly enhances the intrinsic PAOR performance (Fig. 3a and Fig. S13). Whether using β-Ni(OH)_2_ or S-V_O_-β-Ni(OH)_2_ as the anode, the electrooxidation product of R-CH_2_OH (e.g. ethanol) is R-COOH (e.g. acetic acid), and the PAOR performance of S-V_O_-β-Ni(OH)_2_ remains stable during consecutive electrolysis (Figs S14 and S15). Next, we identified and compared the catalyst functions of PAORs over β-Ni(OH)_2_ and S-V_O_-β-Ni(OH)_2_ to investigate the catalytic role of electrooxidation-induced oxygen vacancies in the PAOR. The PAOR system differs from the OER system, and the morphology and crystal structure of the catalyst remained nearly constant during PAORs over β-Ni(OH)_2_ or S-V_O_-β-Ni(OH)_2_ (Figs S16 and S17). *In-situ* Raman spectra proved that Ni^2+δ^ species (e.g. Ni^3+^–O bonds and Ni^2+δ^–OH_ads_) could not accumulate on the electrode surface during PAORs over β-Ni(OH)_2_ or S-V_O_-β-Ni(OH)_2_ (Figs S16 and S17) [38]. Besides, there are almost no S atoms remaining in the S-V_O_-β-Ni(OH)_2_ electrode after the PAOR, indicating that, during the PAOR over S-V_O_-β-Ni(OH)_2_, electrooxidation-induced oxygen vacancies can be exposed again via the loss of S atoms (Fig. S18 and Table S5).

EIS data show that the PAOR over β-Ni(OH)_2_ only involves the high-frequency step, i.e. the generation of electrophilic lattice oxygen species (Fig. 3b and c, and Fig. S19) [25]. However, in addition to the high-frequency step, the PAOR over S-V_O_-β-Ni(OH)_2_ also involves the low-frequency step, i.e. the generation of electrophilic adsorbed oxygen species (Fig. 3d and Fig. S20). Hence, the only active intermediate of the PAOR on β-Ni(OH)_2_ is the Ni^3+^–O bond containing electrophilic lattice oxygen; however, both electrophilic lattice and adsorbed oxygen species (e.g. Ni^3+^–O bonds and Ni^2+δ^–OH_ads_) are active intermediates of the PAOR over S-V_O_-β-Ni(OH)_2_. The difference between frequency dependencies of the PAOR over β-Ni(OH)_2_ and S-V_O_-β-Ni(OH)_2_ indicates that S-V_O_ plays a critical role in the generation of electrophilic adsorbed oxygen species. Adsorbed oxygen species (e.g. OH_ads_) is usually adsorbed on coordinatively unsaturated metal sites [39,40]. The surface of the β-Ni(OH)_2_ nanosheet is predominantly covered with lattice hydroxyl groups, leaving few exposed coordinatively unsaturated Ni atoms; hence, OH_ads_ can hardly be directly adsorbed on the surface of β-Ni(OH)_2_ [41]. On the other hand, during the PAOR over S-V_O_-β-Ni(OH)_2_, coordinatively unsaturated Ni atoms adjacent to S-V_O_ can electrochemically adsorb hydroxyl ions to generate OH_ads_ (S-V_O_-Ni^2+δ^-OH_ads_), thus leading to the low-frequency electrochemical step.

To identify the catalyst function, it is crucial to decouple the reaction between active intermediates and alcohols from the PAOR. Via combining multi-potential step chronoamperometry measurements and *in-situ* Raman spectra, we analyzed the catalytic role of electrophilic oxygen species in the PAOR (Figs S21–S24). Ni^3+^–O bonds can be generated and accumulated on the β-Ni(OH)_2_ electrode at the oxidation potential in 1 M KOH, and accumulated Ni^3+^–O bonds can spontaneously react with alcohol to generate organic oxidation products and Ni^2+^–OH bonds under open circuit conditions (Figs S21 and S22). Consequently, the dehydrogenation of alcohols catalyzed by Ni^3+^–O bonds is a spontaneous catalyst-induced non-electrochemical step, and it is defined as lattice oxygen-induced hydrogen atom transfer (LO-HAT) (Fig. 3c). In general, the PAOR over β-Ni(OH)_2_ is an indirect electrooxidation reaction with Ni^3+^–O bonds containing electrophilic lattice oxygen as the redox mediator. As to PAORs over β-Ni(OH)_2_, the catalyst function follows a lattice oxygen-mediated mechanism involving HAT (LOM-HAT), including the electrooxidation of Ni^2+^–OH to Ni^3+^–O bond and LO-HAT (Fig. 3c).

On the other hand, both the electrophilic lattice and adsorbed oxygen species (e.g. Ni^3+^–O bonds and S-V_O_-Ni^2+δ^-OH_ads_) can be generated and accumulated on the S-V_O_-β-Ni(OH)_2_ electrode surface during electrooxidation in 1 M KOH (Fig. S23). Besides, accumulated Ni^2+δ^ species containing electrophilic lattice/adsorbed oxygen can spontaneously react with alcohols to generate carboxylic acid and Ni^2+^ species under open-circuit conditions (Fig. 3e, and Figs S23 and S24). Thus, in addition to the Ni^3+^–O bonds, S-V_O_-Ni^2+δ^-OH_ads_ can spontaneously catalyze the dehydrogenation of alcohols as well, and this catalyst-induced non-electrochemical step is defined as S-V_O_-induced adsorbed oxygen-induced hydrogen atom transfer (S-V_O_-AO-HAT). The PAOR over S-V_O_-β-Ni(OH)_2_ is an indirect electrooxidation reaction with electrophilic lattice and adsorbed oxygen species (i.e. Ni^3+^–O bonds and S-V_O_-Ni^2+δ^-OH_ads_) as the redox mediator. Consequently, in addition to LOM-HAT, the PAOR over S-V_O_-β-Ni(OH)_2_ also follows S-V_O_-induced adsorbed oxygen-mediated HAT (S-V_O_-AOM-HAT), including the generation of S-V_O_-Ni^2+δ^-OH_ads_ and S-V_O_-AO-HAT (Fig. 3f).

Considering spontaneous electrophilic oxygen-induced HAT steps (e.g. LO-HAT and S-V_O_-AO-HAT), the rate-determining step for AORs on NiO*_x_*H*_y_* species is the electrochemical step, i.e. the generation of electrophilic oxygen species [42]. According to density functional theory (DFT) calculations, the oxygen vacancy can prompt the dehydrogenation of Ni^2+^–OH to Ni^3+^–O bonds over β-Ni(OH)_2_ (001), and the energy barrier is decreased from 1.43 eV to 1.30 eV (Fig. S25). Hence, S-V_O_ is in favor of LOM-HAT. The generation of S-V_O_-Ni^2+δ^-OH_ads_ requires overcoming a low energy barrier of 0.73 eV so that the thermodynamics of S-V_O_-AOM-HAT is better than that of LOM-HAT (Fig. 3g and Fig. S26). By accelerating the HAT process, S-V_O_ significantly enhances the PAOR performance of S-V_O_-β-Ni(OH)_2_.

S-V_O_-β-Ni(OH)_2_ is appropriate in all kinds of PAOR systems, and S-V_O_-β-Ni(OH)_2_ can effectively catalyze the electrooxidation of R–CH_2_OH to R-COOH (Figs S14 and S27). Nevertheless, the catalyst function involving HAT (e.g. LOM-HAT and S-V_O_-AOM-HAT) cannot explain the oxygen atom transfer in the electrooxidation of R–CH_2_OH to R–COOH. Our previous work proves that, in addition to catalyst functions, the PAOR also involves a spontaneous catalyst-irrelevant non-electrochemical step involving oxygen atom transfer, i.e. the hydration of aldehyde (R–CHO) [43,44]. Specifically, the reaction pathway of the PAOR over S-V_O_-β-Ni(OH)_2_ includes three processes (Fig. 3h and Fig. S28). First, due to LOM-HAT and S-V_O_-AOM-HAT, R–CH_2_OH loses two hydrogen atoms to form R-CHO. Next, the nucleophilic attack of water molecules on R–CHO causes the formation of aldehyde hydrates (R–CH(OH)_2_). Eventually, R–CH(OH)_2_ undergoes two HAT processes to generate R–COOH.

### Catalytic role of S-V_O_ in the POR

**Figure 4.**
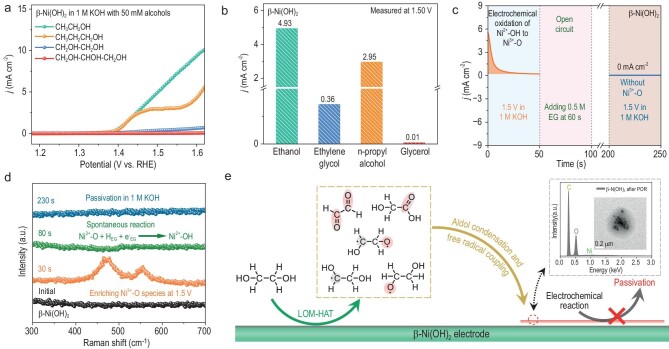
Reaction mechanism of the POR over β-Ni(OH)_2_. (a) Anodic polarization curves of β-Ni(OH)_2_ in the PAOR and POR systems (1 M KOH with 50 mM alcohols; primary alcohols: ethanol and n-propyl alcohol; polyols: ethylene glycol (EG) and glycerol (GLY)). (b) Histogram showing electrochemical performances of PAORs and PORs on β-Ni(OH)_2_ at the potential of 1.50 V. (c) Multi-potential step chronoamperometry of the β-Ni(OH)_2_ electrode in the OER and POR systems. (d) Synchronous *in-situ* Raman spectra of the β-Ni(OH)_2_ electrode during electrochemical testing, which is shown in (c). (e) Schematic diagram of the passivation mechanism of PORs over β-Ni(OH)_2_.

Our previous work has proven that Ni^2+δ^–OH_ads_ containing electrophilic adsorbed oxygen plays a critical role in the C–C bond cleavage during the POR over NiO*_x_*H*_y_* [43]. However, theoretically, the electrophilic lattice oxygen in the Ni^3+^–O bond only acts as a hydrogen acceptor to catalyze HAT processes, instead of the C–C bond cleavage, because of its special coordination structure (Fig. S29) [25]. The only active intermediate of AORs over β-Ni(OH)_2_ is the Ni^3+^–O bond. As a result, β-Ni(OH)_2_ really cannot catalyze the electrooxidation of polyols to FA involving the C–C bond cleavage and shows poor POR performance (Fig. 4a and b). The OER performance of the β-Ni(OH)_2_ electrode after the POR is far worse than that of the fresh β-Ni(OH)_2_ electrode, and the Ni^2+^/Ni^3+^ oxidation peak cannot be observed in its anodic polarization curves (Fig. S30). This indicates that the β-Ni(OH)_2_ electrode can be irreversibly passivated during the POR.

A combination of multi-potential step chronoamperometry and *in-situ* Raman measurements proved the irreversible passivation of β-Ni(OH)_2_ in the POR system (Fig. 4c and d). Under open circuit conditions, accumulated Ni^3+^–O bonds on the β-Ni(OH)_2_ electrode disappeared completely as soon as polyol (e.g. EG) was added into the electrolyte, proving that Ni^3+^–O bonds can spontaneously catalyze the dehydrogenation of polyols via LOM-HAT. However, as to the β-Ni(OH)_2_ electrode after the POR, Ni^2+^–OH bonds could not be electrochemically oxidized to Ni^3+^–O bonds under an oxidation potential of 1.5 V (Fig. 4e). Besides, TEM images show that the β-Ni(OH)_2_ nanosheets after the POR were coated by polymers (Fig. S31 and Table S6). These results indicate that, for the POR over β-Ni(OH)_2_, polyols can be electrochemically oxidized to polymers, which are attached to the electrode surface as an insoluble passivation film.

The only catalyst function, i.e. LOM-HAT, must play an important role in the passivation mechanism of PORs over β-Ni(OH)_2_. Hence, the reaction intermediates of the POR must be dehydrogenation products of polyols, such as carbon/oxygen radicals and carbonyl compounds. During the passivation of β-Ni(OH)_2_ in the POR, the dehydrogenation intermediates should undergo spontaneous coupling reactions to generate long-chain compounds. There are two possible spontaneous coupling reactions between dehydrogenation intermediates, i.e. radical coupling and condensation reactions [45–48]. Hence, PORs over β-Ni(OH)_2_ may involve the following two catalyst-irrelevant non-electrochemical steps: (i) radical coupling between carbon/oxygen radicals and (ii) condensation between carbonyl compounds. Due to the synergy of LOM-HAT and spontaneous coupling reactions between dehydrogenation intermediates for PORs over β-Ni(OH)_2_, polyols can be electrochemically oxidized to form a passivation film containing polymers on the electrode surface, and the insulating passivation film hinders the contact between electrode and electrolytes, thus resulting in electrode passivation (Fig. 4e and Fig. S32).

**Figure 5.**
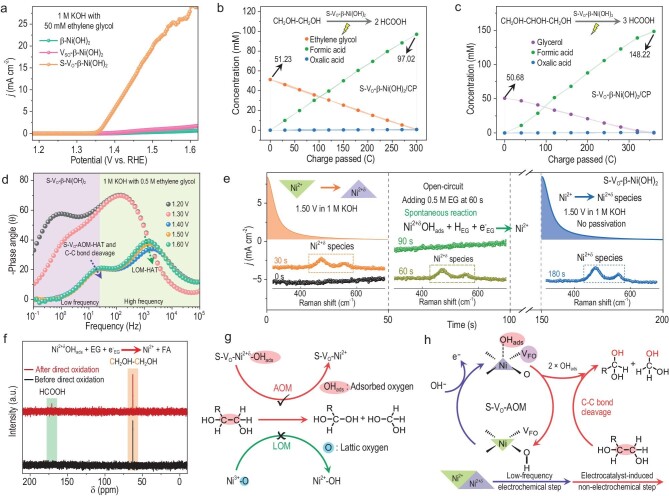
Catalyst function involving C–C bond cleavage of S-V_O_-β-Ni(OH)_2_ in the POR. (a) Anodic polarization curves comparing β-Ni(OH)_2_, V_SO_-β-Ni(OH)_2_ and S-V_O_-β-Ni(OH)_2_ in the model POR system (1 M KOH with 50 mM EG). (b, c) Concentration changes of polyols and their electrooxidation products versus charge passed during PORs over S-V_O_-β-Ni(OH)_2_, including EG electrooxidation (b) and GLY electrooxidation (c). (d) Bode plots of the POR over S-V_O_-β-Ni(OH)_2_ at different potentials (1 M KOH with 0.5 M EG). (e) Multi-potential step chronoamperometry and synchronous *in-situ* Raman spectra of the S-V_O_-β-Ni(OH)_2_ electrode in the OER and POR systems. (f) ^13^C nuclear magnetic resonance (NMR) spectra of the electrolyte (1 M KOH with 50 mM EG) before/after 20 reaction cycles with Ni^2+δ^ species. Detailed information is shown in Fig. S39. (g) Schematic diagram illustrating the active intermediate for the C–C bond cleavage. (h) Schematic diagram showing S-V_O_-AOM-Cleavage of the C–C bond, comprising the electrochemical generation of S-V_O_-Ni^2+δ^-OH_ads_ and S-V_O_-AO-Cleavage of the C–C bond.

Theoretically, the β-Ni(OH)_2_ with rich oxygen vacancies should exhibit excellent POR performance, because both the HAT and the C–C bond cleavage can be catalyzed by the oxygen-vacancy-induced electrophilic adsorbed oxygen species [3]. However, similar to PORs over β-Ni(OH)_2_, V_SO_-β-Ni(OH)_2_ could be passivated during PORs, proving that inactivated oxygen vacancies in V_SO_-β-Ni(OH)_2_ cannot drive the oxygen-vacancy-induced catalytic mechanism (Fig. 5a and Figs S33 and S34). Miraculously, S-V_O_-β-Ni(OH)_2_ exhibits excellent POR performance, and the electrode passivation cannot be observed during PORs over S-V_O_-β-Ni(OH)_2_ (Fig. 5a, Figs S35 and S36 and Table S7). Based on product analysis of PORs over S-V_O_-β-Ni(OH)_2_, the electrooxidation product of polyols (e.g. EG and GLY) is FA, and the C–C bond cleavage is involved in those reaction pathways (Fig. 5b and c). In the POR system based on S-V_O_-β-Ni(OH)_2_, one CH_2_OH–CH_2_OH or CH_2_OH–CHOH–CH_2_OH molecule can be electrochemically oxidized to two molecules of HCOOH or three molecules of HCOOH, respectively [11,13]. Moreover, based on S-V_O_-β-Ni(OH)_2_, vicinal diol (R–CHOH–CH_2_OH) containing two hydroxyl groups can be electrochemically oxidized to produce R–COOH and HCOOH, accompanied by cleavage of the C–C bond (Fig. S37).

EIS data indicated that, similar to PAORs over S-V_O_-β-Ni(OH)_2_, both the high-frequency and low-frequency steps, i.e. the generation of the electrophilic lattice and adsorbed oxygen species, function during PORs over S-V_O_-β-Ni(OH)_2_ (Fig. 5d and Fig. S38). Hence, both Ni^3+^–O bonds and S-V_O_-Ni^2+δ^-OH_ads_ are key active intermediates for PORs over S-V_O_-β-Ni(OH)_2_. According to multi-potential step chronoamperometry *in-situ* Raman measurements and product characterizations, we proved that, on the S-V_O_-β-Ni(OH)_2_ electrode, accumulated Ni^2+δ^ species including Ni^3+^–O bonds and S-V_O_-Ni^2+δ^-OH_ads_ could spontaneously catalyze the oxidative C–C bond cleavage of polyols to generate HCOOH and Ni^2+^ species without the electrode passivation (Fig. 5e and f, and Fig. S39). Given that the Ni^3+^–O bond is unavailable for the C–C bond cleavage, only S-V_O_-Ni^2+δ^-OH_ads_ can spontaneously catalyze the C–C bond cleavage, and this spontaneous catalyst-induced non-electrochemical step is defined as S-V_O_-induced adsorbed oxygen-induced cleavage of C–C bond (S-V_O_-AO-Cleavage of C–C bond) (Fig. 5g). Consequently, in addition to LOM-HAT and S-V_O_-AOM-HAT, PORs over S-V_O_-β-Ni(OH)_2_ also follow S-V_O_-induced adsorbed oxygen-mediated the C–C bond cleavage (S-V_O_-AOM-Cleavage of C–C bond), including the generation of S-V_O_-Ni^2+δ^-OH_ads_ and S-V_O_-AO-Cleavage of C–C bonds (Fig. 5h and Fig. S40). After 24 hours of air exposure, and after sulfur leaching, S-V_O_-β-Ni(OH)_2_ also exhibits excellent POR performance, proving that S-V_O_ is relatively stable under ambient atmosphere (Fig. S35).

### Reaction pathway of the POR on S-V_O_-β-Ni(OH)_2_

**Figure 6.**
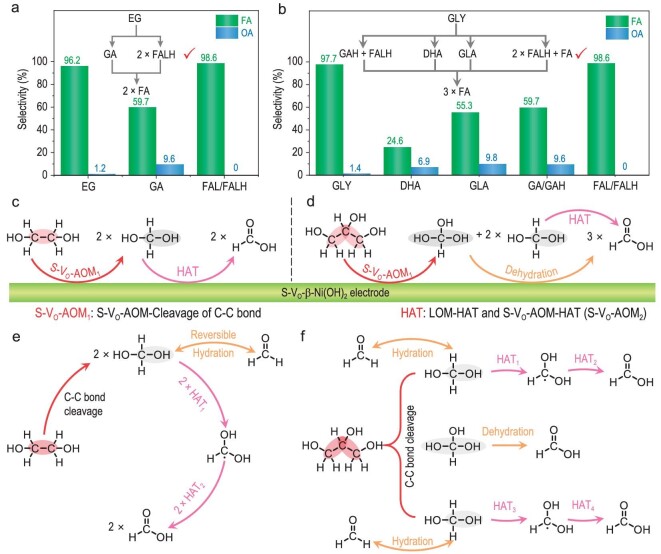
Reaction pathways of PORs on S-V_O_-β-Ni(OH)_2_. (a, b) Selectivities of FA and oxalic acid (OA) for the electrooxidation of possible reaction intermediates in (a) EGOR over S-V_O_-β-Ni(OH)_2_ and (b) GOR over S-V_O_-β-Ni(OH)_2_. (c, d) Schematic diagrams illustrating the reaction pathways for (c) EGOR and (d) GOR on S-V_O_-β-Ni(OH)_2_. (e, f) Reaction pathways of non-electrochemical steps in (e) EGOR and (f) GOR on S-V_O_-β-Ni(OH)_2_.

Although there has been quite a lot of research into PORs over nickel-based catalysts, the detailed reaction pathway of the POR is still unknown [10,49]. As to AORs (including PAORs and PORs) over nickel-based catalysts, the reaction pathway depends on non-electrochemical steps instead of electrochemical steps, because electrochemical steps have no immediate bearing on polyol substrate. PORs over S-V_O_-β-Ni(OH)_2_ may involve three kinds of non-electrochemical steps: (i) electrophilic oxygen-induced HAT (i.e. LO-HAT and S-V_O_-AO-HAT), (ii) S-V_O_-AO-Cleavage of the C–C bond, and (iii) hydration of R-CHO (Fig. S40). We carried out a quantitative analysis of products for the electrooxidation of possible reaction intermediates based on S-V_O_-β-Ni(OH)_2_ to identify key reaction intermediates in PORs (Fig. 6a and b).

As to the EG electrooxidation reaction (EGOR), there are two possible reaction intermediates: (i) formaldehyde hydrate (FALH) generated due to the C–C bond cleavage of EG, and (ii) glycolic aldehyde (GA) generated due to the dehydrogenation of EG (Fig. S41) [11,50]. According to the quantitative analysis, the HCOOH selectivities for the electrooxidation of EG, GA and FALH over S-V_O_-β-Ni(OH)_2_ are 96.2%, 59.7% and 98.6%, respectively (Fig. 6a and Fig. S42). That is, the key reaction intermediate for EGOR over S-V_O_-β-Ni(OH)_2_ is FALH, instead of GA. Therefore, the EGOR pathway based on S-V_O_-β-Ni(OH)_2_ includes two processes: (i) the C–C bond cleavage of EG to generate two molecules of FALH due to the S-V_O_-AOM-Cleavage of the C–C bond, and (ii) the dehydrogenation of FALH to HCOOH due to LOM/S-V_O_-AOM-HAT (Fig. 6c).

As to the glycerol electrooxidation reaction (GOR), there are four possibilities for the first step: (i) the dehydrogenation of hydroxymethyl to generate glyceraldehyde (GLA), (ii) the dehydrogenation of secondary hydroxyl to generate dihydroxyacetone (DHA), (iii) the cleavage of one C–C bond to generate glycolic aldehyde hydrate (GAH) and FALH, and (iv) the cleavage of two C–C bonds to generate one HCOOH and two FALH molecules (Fig. S43) [51–53]. According to the quantitative analysis, only the HCOOH selectivity for FALH electrooxidation (98.6%) is higher than that for GOR (97.7%) while the HCOOH selectivities for the electrooxidation of other possible reaction intermediates are <60% (Fig. 6b and Fig. S44). That is, the key intermediate for GOR over S-V_O_-β-Ni(OH)_2_ is FALH, instead of other multiple-carbonyl organic compounds (Fig. 6d). Therefore, the GOR pathway based on S-V_O_-β-Ni(OH)_2_ includes two processes: (i) the cleavage of two C–C bonds in glycerol to generate one molecule of HCOOH and two molecules of FALH due to S-V_O_-AOM-Cleavage of the C–C bond, and (ii) the dehydrogenation of FALH to HCOOH due to LOM/S-V_O_-AOM-HAT (Fig. 6d).

Bond dissociation free energy (BDFE) can be used to evaluate the ease of chemical bond cleavage [54,55]. The dehydrogenation of FALH to HCOOH is an important process in PORs over S-V_O_-β-Ni(OH)_2_, and it includes two LOM/S-V_O_-AOM-HAT steps. According to DFT calculations, the BDFEs of C–H and O–H bonds in FALH are 92.7 and 96.4 kcal mol^−1^, respectively (Fig. S45). Hence, the dehydrogenation of C–H bond is preferred to the dehydrogenation of O–H bond in the dehydrogenation of FALH to HCOOH. All in all, the detailed POR pathway was revealed by combining experimental data and theoretical calculations. For PORs over S-V_O_-β-Ni(OH)_2_, the first step is the oxidative cleavage of the C–C bond in polyols based on S-V_O_-AOM-Cleavage of C–C bond, and then C–H and O–H bonds in FALH are dehydrogenized in turn to generate HCOOH due to LOM/S-V_O_-AOM-HAT (Fig. 6e and f).

According to the above results, due to the synergy of LOM-HAT, S-V_O_-AOM-HAT and S-V_O_-AOM-Cleavage of C–C bond, S-V_O_-β-Ni(OH)_2_ is very efficient for the electrooxidation of polyols to FA. The S-V_O_-induced catalytic mechanism plays a critical role in PORs, especially for the C–C bond cleavage process. Inspired by this, we used other heteroatoms (e.g. N and P) to fill oxygen vacancies to synthesize oxygen-vacancy-filling with other heteroatoms in β-Ni(OH)_2_, e.g. N-V_O_-β-Ni(OH)_2_ and P-V_O_-β-Ni(OH)_2_ (Fig. S46). Both N-V_O_-β-Ni(OH)_2_ and P-V_O_-β-Ni(OH)_2_ show excellent POR performance without the passivation of electrodes (Fig. S46 and Table S8). These results validate the general effectiveness of this POR-catalyst design strategy of filling oxygen vacancies with removable heteroatoms.

## CONCLUSION

AORs over NiO*_x_*H*_y_* are indirect electrooxidation reactions with electrophilic lattice/adsorbed oxygen species (i.e. Ni^3+^–O bonds or Ni^2+δ^–OH_ads_) as the redox mediator. The generation of Ni^2+δ^–OH_ads_ depends on oxygen vacancies. Both Ni^3+^–O bonds and Ni^2+δ^–OH_ads_ can spontaneously catalyze the HAT process, e.g. the dehydrogenation of alcohols; however, the C–C bond cleavage can be catalyzed by Ni^2+δ^–OH_ads_, instead of Ni^3+^–O bonds. Hence, the oxygen-vacancy-induced adsorbed oxygen-mediated mechanism (V_O_-AOM) is essential so that the electrochemical POR can generate FA involving the C–C bond cleavage. However, it is easy for the high-energy Schottky-type oxygen vacancies in V_SO_-β-Ni(OH)_2_ to be deactivated. As a result, both β-Ni(OH)_2_ and V_SO_-β-Ni(OH)_2_ are not suitable for PORs because the only catalyst function of LOM-HAT is ineffective for C–C bond cleavage. As to S-V_O_-β-Ni(OH)_2_, filling S atoms protects unstable oxygen vacancies. During PORs over S-V_O_-β-Ni(OH)_2_, the pre-electrooxidation-induced structural reconstruction causes the *in-situ* generation of stable Frenkel-type oxygen vacancies, which guarantee the sustained effect of oxygen-vacancy-induced catalytic mechanisms, e.g. S-V_O_-AOM-HAT and S-V_O_-AOM-Cleavage of C–C bond. Consequently, S-V_O_-β-Ni(OH)_2_ can effectively catalyze the electrooxidation of polyols to FA, and shows excellent POR performance without the electrode passivation. This work reveals the S-V_O_-induced catalytic process in AORs (including the PAOR and the POR) over S-V_O_-β-Ni(OH)_2_, which provides important theoretical guidance for the highly effective POR catalyst design strategy. More importantly, this intelligent catalyst design strategy of filling oxygen vacancies with heteroatoms offers unlimited scope for ensuring the sustained action of the oxygen-vacancy-induced catalytic mechanism during electrooxidation reactions.

## METHODS

### Preparation of β-Ni(OH)_2_

Briefly, 20 mmol of Ni(NO_3_)_2_·6H_2_O was dissolved in 40 mL of deionized water under vigorous stirring for 10 min to form a homogeneous solution. Next, 40 mL of NaOH solution (2 M) was added to the above solution. The mixture was transferred into a 100-ml Teflon-lined autoclave, maintained at 160°C for 6 h and cooled down naturally. The product was collected as a turquoise solid by filtration and washed thoroughly with deionized water.

### Preparation of S-V_O_-β-Ni(OH)_2_ and V_SO_-β-Ni(OH)_2_

β-Ni(OH)_2_ nanosheets were placed in the quartz tube posterior continuous cooling plasma zone. Ar was used as a carrier gas (5 sccm) to transport sulfur ions across the plasma zone. When the sulfur source was heated to 200°C, the β-Ni(OH)_2_ nanosheets were treated by plasma (13.56 MHz RF) with different irradiation times (0, 1, 2, 3, 4 and 5 min) at 200 W and 150 Pa. The treated β-Ni(OH)_2_ (for 5 min) was used for detailed study. V_SO_-β-Ni(OH)_2_ was prepared by the same method but without sublimed sulfur. The V_SO_-β-Ni(OH)_2_ catalyst was treated with Ar plasma for 5 min.

### Preparation of P-V_O_-β-Ni(OH)_2_

β-Ni(OH)_2_ nanosheets were placed in the quartz tube posterior continuous cooling plasma zone. Ar was used as a carrier gas (5 sccm) to transport phosphorus ions across the plasma zone. When the phosphorus source was heated to 250°C, the β-Ni(OH)_2_ nanosheets were treated by plasma (13.56 MHz RF) with irradiation for 5 min at 200 W and 150 Pa.

### Preparation of N-V_O_-β-Ni(OH)_2_

β-Ni(OH)_2_ nanosheets were placed in the quartz tube posterior continuous cooling plasma zone. β-Ni(OH)_2_ nanosheets were treated with plasma (13.56 MHz RF), with irradiation for 5 min at 200 W and 150 Pa with NH_3_ as the nitrogen source.

## Supplementary Material

nwae271_Supplemental_File
