# Supplementary material for: Sulfur filling activates vacancy-induced C–C bond cleavage in polyol electrooxidation
Source: Natl Sci Rev. 2024 Aug 5;11(10):nwae271. doi: 10.1093/nsr/nwae271 (PMC11409883; doi:10.1093/nsr/nwae271)
Supplement: nwae271_Supplemental_File [file nwae271_supplemental_file.pdf]

Supporting Information

Sulfur filling activates vacancy-induced C-C bond cleavage in the polyols electrooxidation

Jianqiao Shi[1,†], Wei Chen[1,†,*], Yandong Wu[1], Yanwei Zhu[1], Chao Xie[1], Yimin Jiang[1], Yu-Cheng Huang[2], Chung-Li Dong[2], and Yuqin Zou[1,*]

[1]State Key Laboratory of Chemo/Bio-Sensing and Chemometrics, College of Chemistry and Chemical Engineering, Advanced Catalytic Engineering Research Center of the Ministry of Education, Hunan University, Changsha 410082
[2]Research Center for X-ray Science & Department of Physics, Tamkang University, New Taipei City 25137
***Corresponding authors.** E-mails: weichen1@hnu.edu.cn; yuqin_zou@hnu.edu.cn
†Equally contributed to this work.

Methods

Reagents. Most inorganic compounds were purchased from Sinopharm Chemical Reagent Co., Ltd and Adamas-beta, Shanghai Titan Chemical Co., Ltd, including S_8, NaH_2PO_2, KOH, NaOH, $Ni(NO_3)_2$ $6H_2O$, *etc.* Nafion solution (5 wt%) was purchased from DuPont™. All organic compounds were purchased from Sigma-Aldrich. Deionized water was used in all experiments.

Materials characterization. Transmission electron microscopy (TEM, JEM-2100F) and scanning electron microscopy (SEM, ZEISS Sigma 300) were used to examine the morphology and microstructure of electrocatalysts. The X-ray powder diffraction (XRD) patterns were obtained with a Siemens D500 powder diffractometer using Cu-Kα radiation (λ = 1.54056 Å). X-ray photoelectron spectroscopy (XPS) measurements were carried out with Axis Supra spectrometer. Inductively coupled plasma mass spectrometry (ICP-MS) analysis was tested with Agilent ICP-MS 7800. Electron paramagnetic resonance (EPR) spectra were recorded with a JEOL JES-FA200 spectrometer at 77 K. X-ray absorption near-edge structure (XANES) and Extended X-ray absorption fine structure (EXAFS) were recorded at TPS 44A of National Synchrotron Radiation Research Center, Taiwan. Nuclear magnetic resonance (NMR) spectra were recorded on Bruker AVANCE III 400 (400 MHz) spectrometer. High-performance liquid chromatography (HPLC) was carried out on a Shimadzu LC-20A HPLC system.

Electrochemical measurements. We used the drop coating method to drop the catalyst on the surface of a glassy carbon (GC) electrode with a diameter of 5 mm. Dispersing 4 mg catalyst to the mixture solution (450 µL of deionized water, 500 µL of ethanol, and 50 µL 5% Nafion), the catalyst ink was successfully prepared after sonication for 20 min. 10 µL of the homogeneous catalyst ink was dropped on a GC electrode, and the catalyst loading was 0.2 mg cm^{-2}. The electrode was dried at ambient temperature. A three-electrode configuration was formed with a Hg/HgO reference electrode and a carbon rob counter electrode. Potentials converted to reversible hydrogen electrode (RHE) reference scale via the Nernst equation: $E_{RHE} = E_{Hg/HgO} + 0.098 + 0.059 \times pH$. Electrochemical measurements were performed using an electrochemical workstation (CHI760E, CH Instruments). Polarization curves were completed at 5 mV s^{-1} scan rate. All polarization curves (unless explicitly stated) were obtained after at least three cycles. To improve the current efficiency during the conversion of organic substrate, the catalyst ink was coated on carbon paper (CP).

In situ EIS measurements were carried out in three-electrode potentiostat (Autolab PGSTAT302N) with the frequency ranging from 10^{-2} to 10^5 Hz with an amplitude of 10 mV. For OER system, the electrolyte is 1 M KOH; For PAOR system, the electrolyte is 1 M KOH with 0.5 M primary alcohol; For POR system, the electrolyte is 1 M KOH with 0.5 M polyol.

In situ Raman spectrum was performed on a confocal Raman microscope (Alpha300R, WITEC, Germany, 532 nm laser) at different potentials using a CHI 760E electrochemical workstation. All electrochemical tests were performed with a three-electrode configuration. The electrolyte system was the same as that in the *in situ* EIS measurements.

For the characterization of electrodes (such as SEM, TEM, Raman, and XPS spectra), the glassy carbon plate electrode (1×2 cm^2) was used, and a Nafion-free electrocatalyst ink (consisting of 4 mg electrocatalyst, 500 µL deionized water, and 500 µL ethanol) was adopted to prevent the effect of Nafion on these characterizations. The potential of electrolysis, electrolysis times, and concentration of alcohol are 1.45 V_{RHE}, one hour, and 0.5 M, respectively.

To facilitate the conversion of the substrate, 50 mM alcohol was selected for the electrolysis process. For the identification and quantification of reaction intermediates and products in AORs, the catalyst was painted on carbon paper (CP; 1 cm^2) in order to increase the electrolysis efficiency of AORs.

Products analysis. The substrates and the products were quantified using HPLC equipped with a refractive detector and a 7.8 × 300 mm Coregel-87H3 column. Column temperature was kept at 60 ℃ and mobile phase was sulfuric acid 5 mM with a flow rate of 0.5 mL min^{-1}. After electrolysis, 50 μL of the electrolyte was taken out, and its pH was adjusted to neutral by adding 0.5 M H_2SO_4. The filtered sample was injected into the HPLC for retention time (30 min) analysis. Organic compounds were characterized by ^1H NMR and ^{13}C NMR. Typically, 400 μL of electrolyte after electrolysis was mixed with 200 μL of D_2O.

The conversion rate, selectivity, and Faradic efficiency are calculated as follows:

Conversion rate (%) = (mol of nucleophile consumed) / (mol of the nucleophile at the beginning) × 100%

Selectivity (%) = (mol of product) / (mol of consumed nucleophile) × 100%

Faradic efficiency (%) = (mol of product) × n × F / (total passed charge) × 100%

n = 2 for the electrooxidation of R-CH_2OH to R-CHO, R-CHO/R-CH$(OH)_2$ to R-COOH, and the cleavage of C-C bond.

n = 4 for the electrooxidation of R-CH_2OH to R-COOH.

n = 6 for the electrooxidation of R-CHOH-CH_2OH to R-COOH and HCOOH.

n = 8 for the electrooxidation of CH_2OH-CHOH-CH_2OH to HCOOH.

F is the Faraday constant (96485 C mol^{-1}).

Theoretical methods. All the spin-polarized calculations were performed within the framework of DFT using the projector-augmented plane wave (PAW) technique as implemented in the Vienna Ab Initio Simulation Package (VASP 5.4) [1]. The generalized gradient approximation (GGA) method parameterized by the Perdew-Burke-Ernzerhof (PBE) functional was used to account for the exchange-correlation energy [2, 3]. The kinetic energy cutoff was set to be 450 eV in the plane-wave expansion. We constructed (2×2) supercells of β-Ni$(OH)_2$ (001) surfaces, one without vacancy and another with oxygen vacancies, each consisting of five atomic layers. The bottom two atomic layers remained fixed, and the topmost three atomic layers and adsorbed species underwent relaxation. To avoid interlayer interaction, the vacuum spacing in a direction perpendicular to the plane of the structure is set to 15 Å. The Brillouin zone was sampled using a 3 × 5 × 1 Monkhorst-Pack *k*-point mesh for geometry optimization calculation. The systems were relaxed until the energy and force reached at 1×10^{-6} eV $cell^{-1}$ and 0.02 eV $Å^{-1}$, respectively. The Gaussian smearing width was set to 0.2 eV. To describe the effect of van der Waals interactions, the DFT-D3 empirical correction method was applied [4]. The free energy for the dehydrogenation (E_H) was calculated according to:

$$E_H = E_{V\text{-}H} + 1/2\ E_{H2} - E_{slab}$$

where $E_{V\text{-}H}$, E_{slab}, and E_{H2} represent energy for relaxed slab after H atom desorption, relaxed slab, and gas phase H_2, respectively.

The free energy for the adsorption energies (E_{ads}) was calculated according to:

$$E_{ads} = E_{slab\text{-}x} - E_{slab} - E_x$$

where E_{slab-x} represents energy for relaxed slab after x molecule adsorption, and E_x represents the energy for gas phase x.

Supplementary Figures.

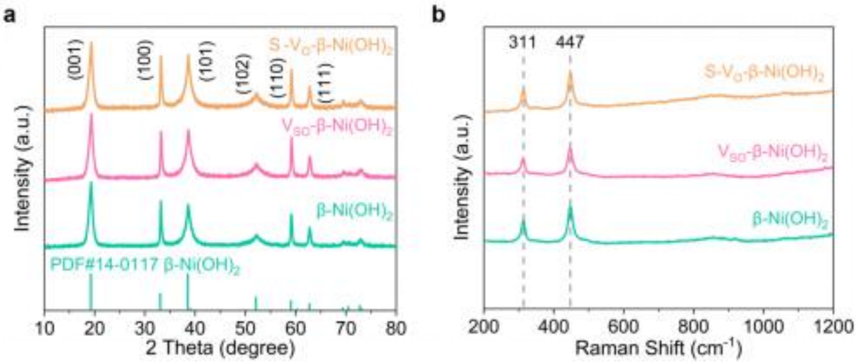

Figure S1 XRD patterns (a) and Raman spectra (b) of β-$Ni(OH)_2$, V_{SO}-β-$Ni(OH)_2$, and S-V_O-β-$Ni(OH)_2$. XRD patterns prove that the crystal phases of V_{SO}-β-$Ni(OH)_2$ and S-V_O-β-$Ni(OH)_2$ are the same as that of β-$Ni(OH)_2$ phase (JCPD card no. 14-0117). The Raman peaks at 311 and 447 cm^{-1} correspond to translational A_{1g}(T) and E_g(T) phonons of β-$Ni(OH)_2$, respectively [5-7]. Clearly, the crystal structures of β-$Ni(OH)_2$, V_{SO}-β-$Ni(OH)_2$, and S-V_O-β-$Ni(OH)_2$ nanosheets are almost identical.

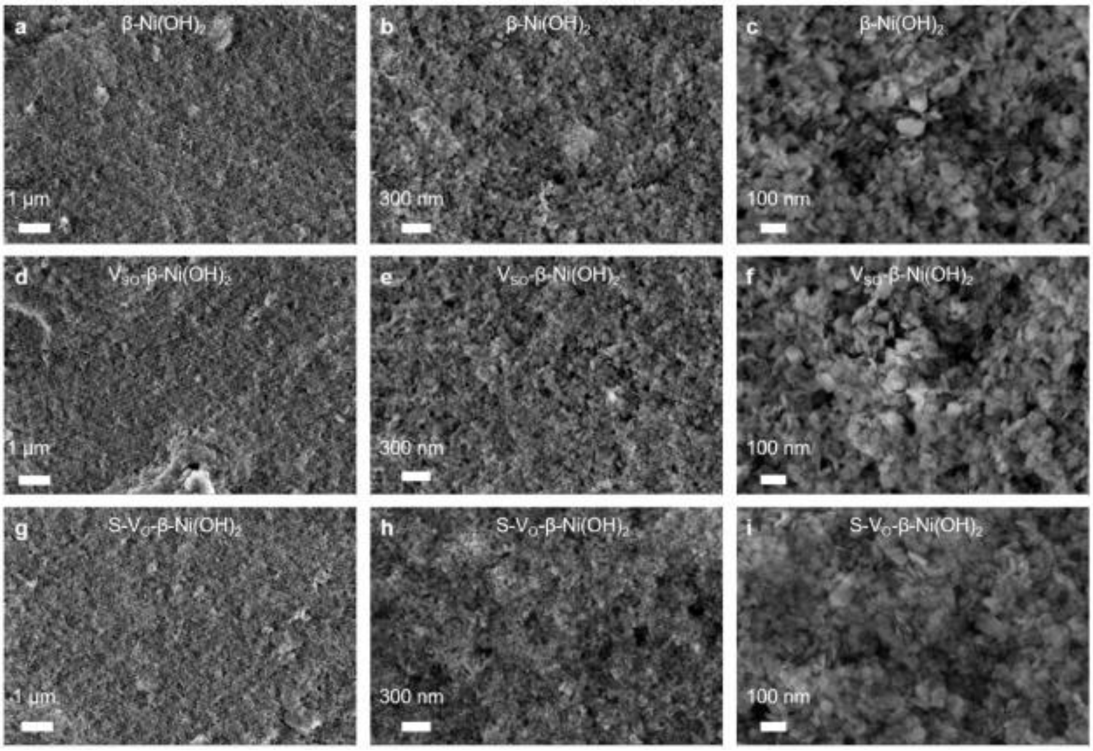

Figure S2 SEM images of β-$Ni(OH)_2$ (a-c), V_{SO}-β-$Ni(OH)_2$ (d-f), and S-V_O-β-$Ni(OH)_2$ (g-i) nanosheets. β-$Ni(OH)_2$, V_{SO}-β-$Ni(OH)_2$, and S-V_O-β-$Ni(OH)_2$ samples are composed of hexagonal nanosheets.

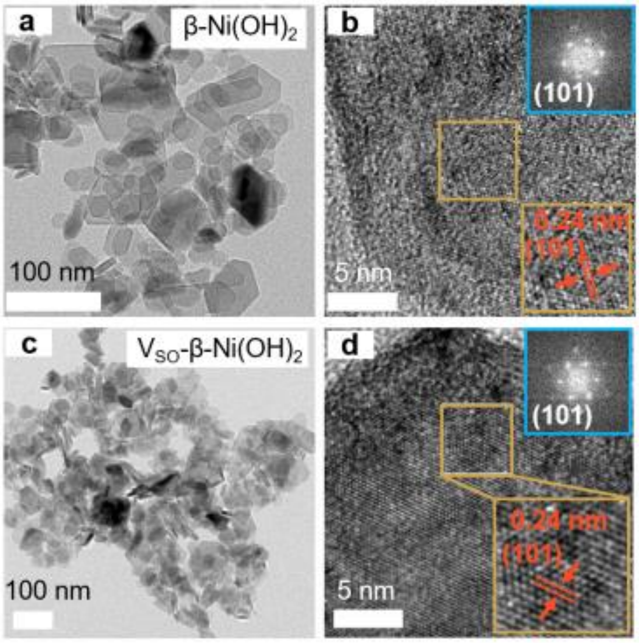

Figure S3 TEM images, HR-TEM images, and SAED patterns of β-$Ni(OH)_2$ (a, b) and V_{SO}-β-$Ni(OH)_2$ (c, d) nanosheets. Both β-$Ni(OH)_2$ and V_{SO}-β-$Ni(OH)_2$ samples consist of hexagonal nanosheets with lateral dimensions in the range of 30-60 nm. The lattice spacing is 0.24 nm, which is consistent with the (101) lattice spacing of β-$Ni(OH)_2$ [8].

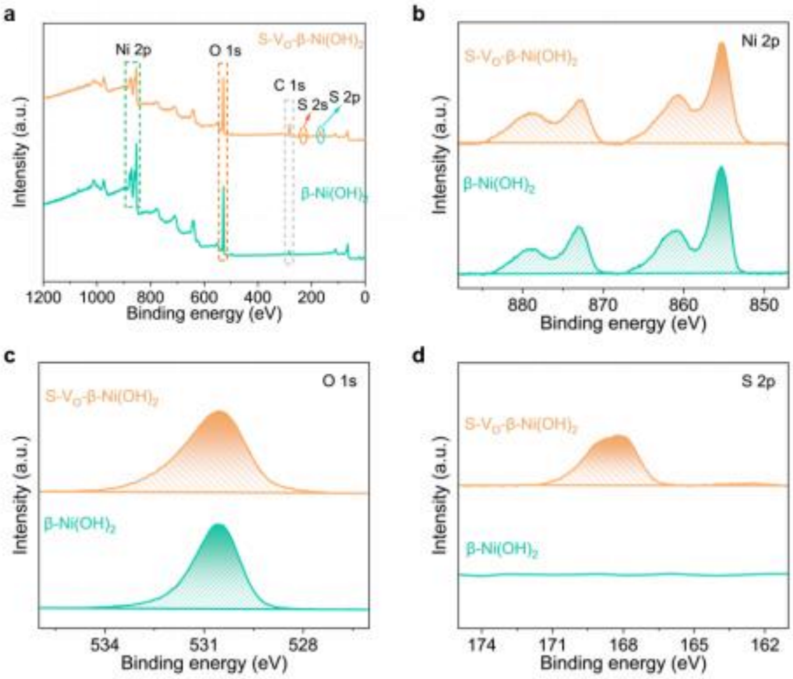

Figure S4 XPS spectra of β-$Ni(OH)_2$ and S-V_O-β-$Ni(OH)_2$. The XPS spectra confirm that S atoms were successfully introduced into the synthesized S-V_O-β-$Ni(OH)_2$.

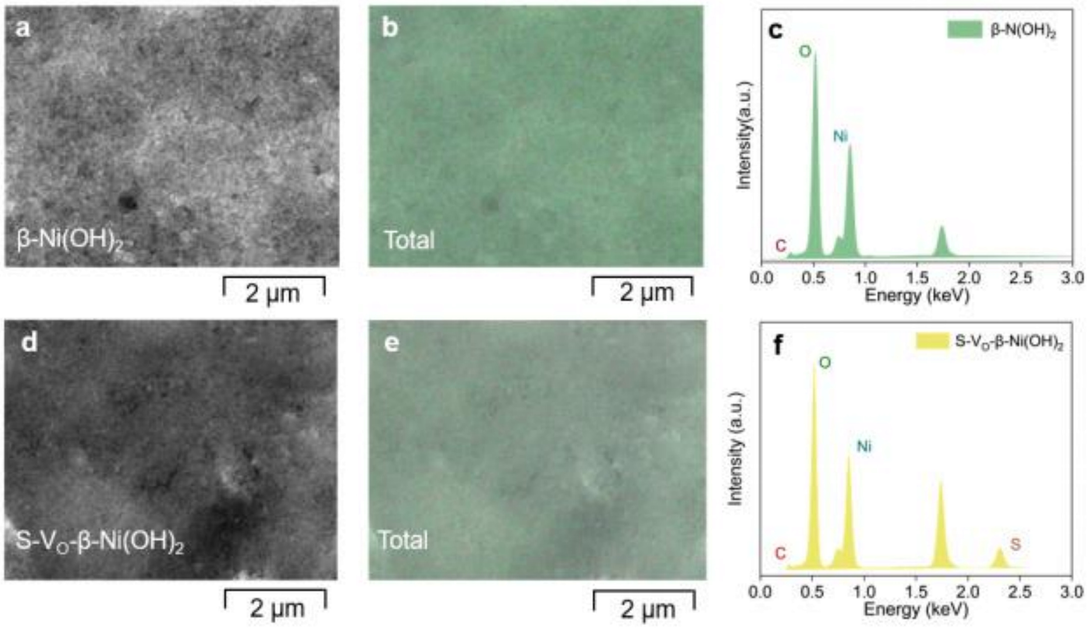

Figure S5 EDX images of β-$Ni(OH)_2$ (a-c) and S-V_O-β-$Ni(OH)_2$ (d-f). The uniform distribution of elemental S inside S-V_O-β-$Ni(OH)_2$ is illustrated by the EDX images.

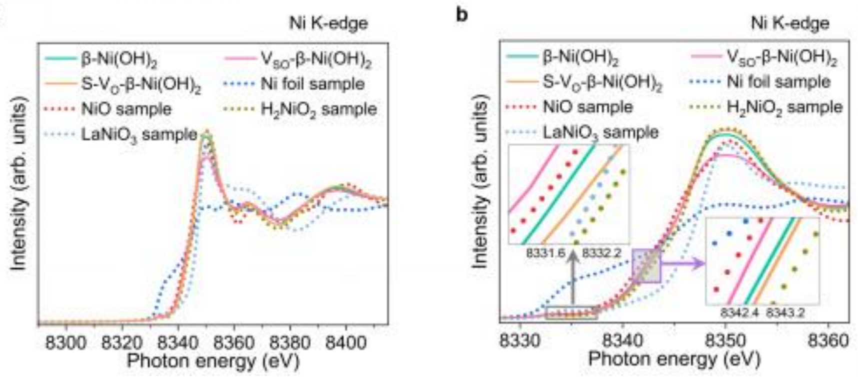

Figure S6 Ni K-edge XANES spectra of β-$Ni(OH)_2$, V_{SO}-β-$Ni(OH)_2$, and S-V_O-β-$Ni(OH)_2$. XANES was measured to determine the electronic and local structures of the samples [9]. Both adsorption edge and pre-edge peak in the Ni K-edge XANES spectra of V_{SO}-β-$Ni(OH)_2$ show obvious peak-shifts toward the low energy regions, compared with that of β-$Ni(OH)_2$. Coordination-unsaturated Ni cations with lower valence states result from oxygen vacancies [10]. The Ni K-edge XANES spectrum of S-V_O-β-$Ni(OH)_2$ reveals that both the adsorption edge and pre-edge peak are shifted back to high energies, indicating that oxygen vacancies have been filled in S-V_O-β-$Ni(OH)_2$ [11].

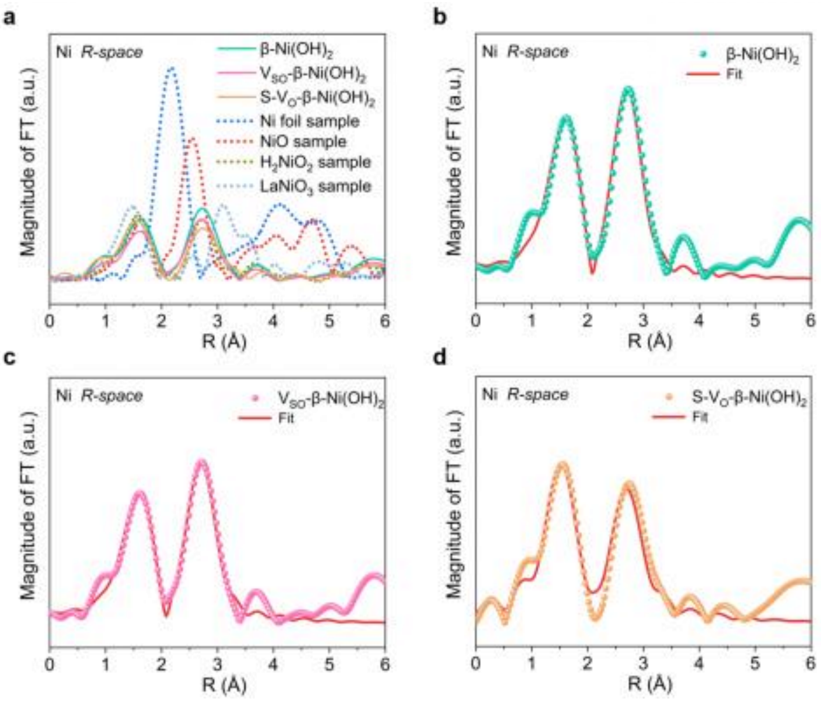

Figure S7 (a) k_3-weighted Fourier transform of EXAFS spectra at Ni K-edge of β-Ni(OH)$_2$, V_{SO}-β-Ni(OH)$_2$, and S-V_O-β-Ni(OH)$_2$. (b-d) k_3-weighted EXAFS spectra (dots) and corresponding fits (lines) in the R space at Ni K-edge of β-Ni(OH)$_2$ (b), V_{SO}-β-Ni(OH)$_2$ (c), and S-V_O-β-Ni(OH)$_2$ (d). Based on EXAFS data acquired at Ni K-edge, we obtained Fourier-transformed EXAFS (FT EXAFS) spectra for analyzing the local coordination environments of nickel atoms in β-Ni(OH)$_2$, V_{SO}-β-Ni(OH)$_2$, and S-V_O-β-Ni(OH)$_2$. Detailed EXAFS fitting results can be found in Table S3.

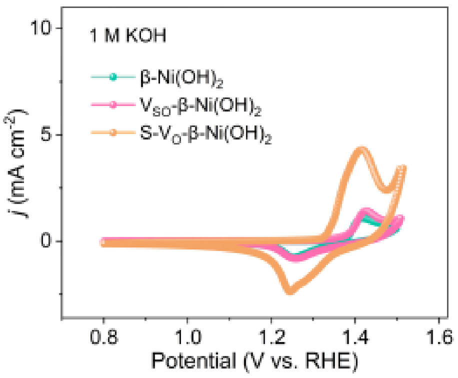

Figure S8 CV curves (5 mV s^{-1}, without iR-correction) of β-Ni(OH)$_2$, V_{SO}-β-Ni(OH)$_2$, and S-V_O-β-Ni(OH)$_2$ on glassy carbon electrode in 1 M KOH. According to cyclic voltammogram (CV) curves, the reduction peak area of S-V_O-β-Ni(OH)$_2$ is about three times those of β-Ni(OH)$_2$ and V_{SO}-β-Ni(OH)$_2$, indicating that S-V_O-β-Ni(OH)$_2$ possesses more Ni active sites than β-Ni(OH)$_2$ and V_{SO}-β-Ni(OH)$_2$. Besides, compared with β-Ni(OH)$_2$ and V_{SO}-β-Ni(OH)$_2$ catalysts, the Ni^{2+}/Ni^{3+} redox peak of S-V_O-β-Ni(OH)$_2$ is significantly shifted to the lower potential. In summary, as to the electrooxidation reaction on S-V_O-β-Ni(OH)$_2$, more Ni active sites could be generated at a lower potential.

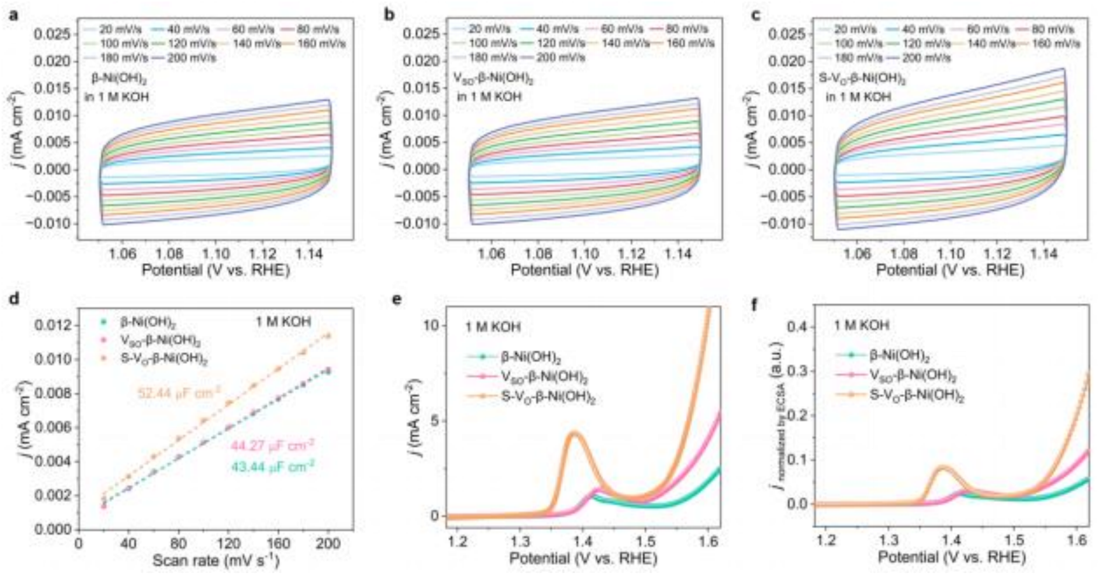

Figure S9 CV curves for β-Ni$(OH)_2$ (a), V_{SO}-β-Ni$(OH)_2$ (b), and S-V_O-β-Ni$(OH)_2$ (c) at different scan rates in 1 M KOH. (d) ECSA values of β-Ni$(OH)_2$, V_{SO}-β-Ni$(OH)_2$ and S-V_O-β-Ni$(OH)_2$. Anodic polarization curves (e) and anodic polarization curves normalized by ECSA (f) of β-Ni$(OH)_2$, V_{SO}-β-Ni$(OH)_2$, and S-V_O-β-Ni$(OH)_2$ in 1 M KOH. The electrochemically active surface area (ECSA) value of S-V_O-β-Ni$(OH)_2$ (52.44 μF cm^{-2}) was slightly higher than those of V_{SO}-β-Ni$(OH)_2$ and β-Ni$(OH)_2$. According to the anodic polarization curves normalized by ECSA, the intrinsic OER activity of S-V_O-β-Ni$(OH)_2$ is significantly better than those of V_{SO}-β-Ni$(OH)_2$ and β-Ni$(OH)_2$.

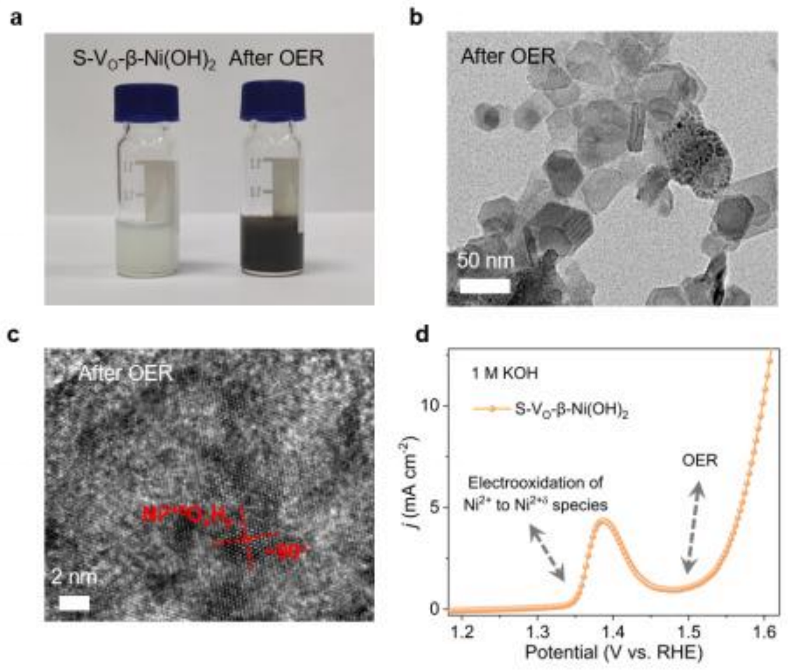

Figure S10 (a) Photograph of fresh S-V_O-β-Ni$(OH)_2$ and the S-V_O-β-Ni$(OH)_2$ after OER suspensions. (b, c) TEM and HR-TEM images of S-V_O-β-Ni$(OH)_2$ nanosheets after OER. (d) Anodic polarization curve of S-V_O-β-Ni$(OH)_2$ in the OER system. After OER, the color of S-V_O-β-Ni$(OH)_2$ changed from turquoise to black due to the formation of black $Ni^{2+\delta}O_xH_y$ species

including NiOOH and NiO_x (Figure S10a) [5]. Meanwhile, the electrooxidation of S-V_O-β-$Ni(OH)_2$ to $Ni^{2+\delta}O_xH_y$ leads to the collapse of regular hexagonal nanosheets. HRTEM images of the S-V_O-β-$Ni(OH)_2$ after OER show distorted lattice fringes with the angle of ~90°, supporting the generation of $Ni^{2+\delta}O_xH_y$ species. Anodic polarization curve of S-V_O-β-$Ni(OH)_2$ in the OER system shows that the Ni^{2+}/Ni^{3+} oxidation peak appears at a potential above about 1.35 V, while the OER reaction occurs at a potential above about 1.50 V.

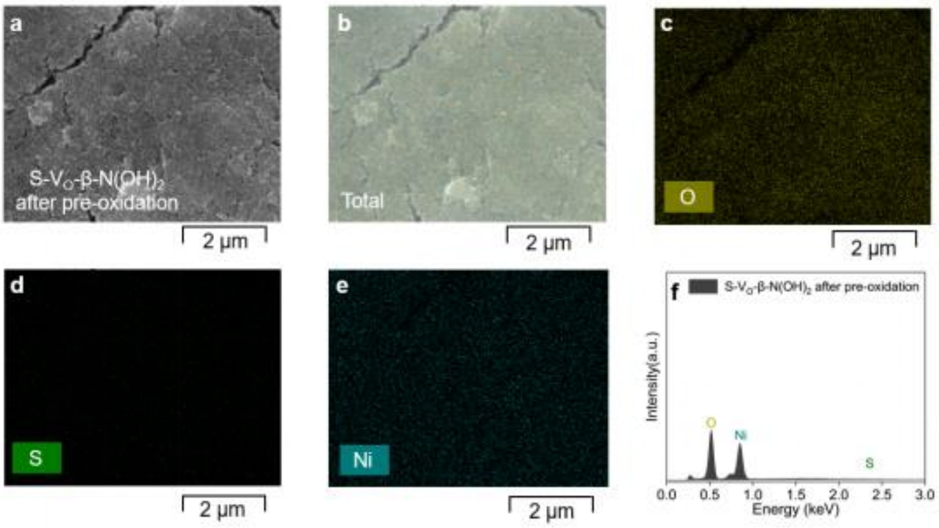

Figure S11 EDX images of the S-V_O-β-$Ni(OH)_2$ electrode after pre-electrooxidation. It proves that, during electrooxidation over S-V_O-β-$Ni(OH)_2$, pre-electrooxidation can cause the irreversible loss of S occurs.

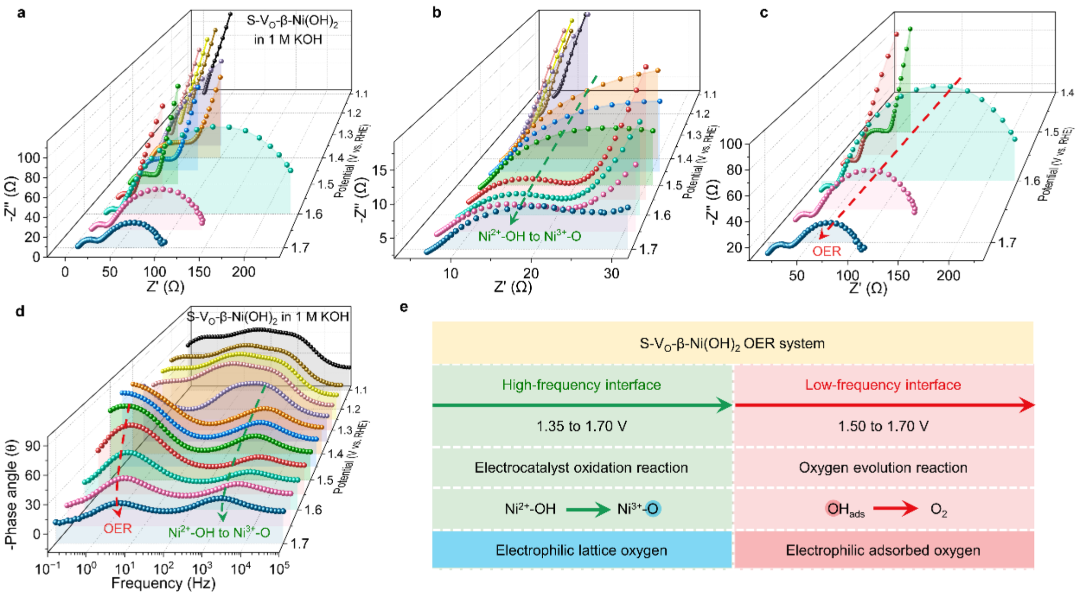

Figure S12 Nyquist plots (a-c), Bode plots (d), and potential-dependent behavior (e) of the S-V_O-β-$Ni(OH)_2$ electrode at different potentials in 1 M KOH. Nyquist plots of S-V_O-β-$Ni(OH)_2$ in 1 M KOH show only one semicircle with a larger radius at the potential below 1.35 V, while two semicircles appear at the potential above 1.35 V (Figure S12a). The radius of the first semicircle (representing the high-frequency electrochemical reaction) decreases gradually at

potentials above 1.35 V (Figure S12b). The radius of the second semicircle (representing the low-frequency electrochemical reaction) significantly decreases at the potential above 1.5 V (Figure S12c). The Bode plot visualizes the different frequency-dependent of these two electrochemical steps (Figure S12d). As to the OER system based on S-V_O-β-$Ni(OH)_2$, the high-frequency electrochemical reaction involves the electrooxidation of Ni^{2+}-OH to Ni^{3+}-O bond containing electrophilic lattice oxygen, and the low-frequency electrochemical reaction is OER involving the generation of electrophilic adsorbed oxygen species (Figure S12e) [12].

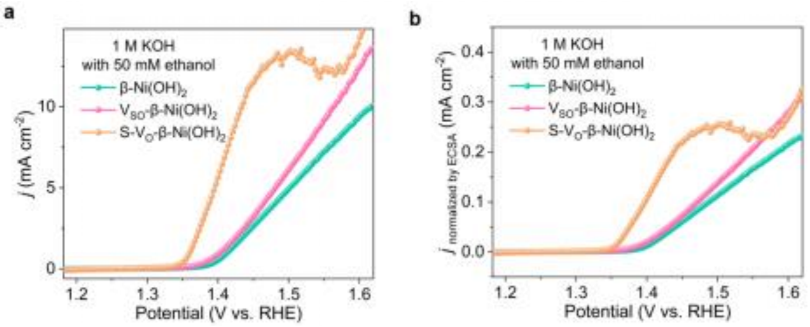

Figure S13 Anodic polarization curves (a) and anodic polarization curves normalized by ECSA (b) of β-$Ni(OH)_2$, V_{SO}-β-$Ni(OH)_2$, and S-V_O-β-$Ni(OH)_2$ in 1 M KOH with 50 mM ethanol. According to anodic polarization curves normalized by ECSA, the intrinsic PAOR activity of S-V_O-β-$Ni(OH)_2$ is significantly better than those of V_{SO}-β-$Ni(OH)_2$ and β-$Ni(OH)_2$.

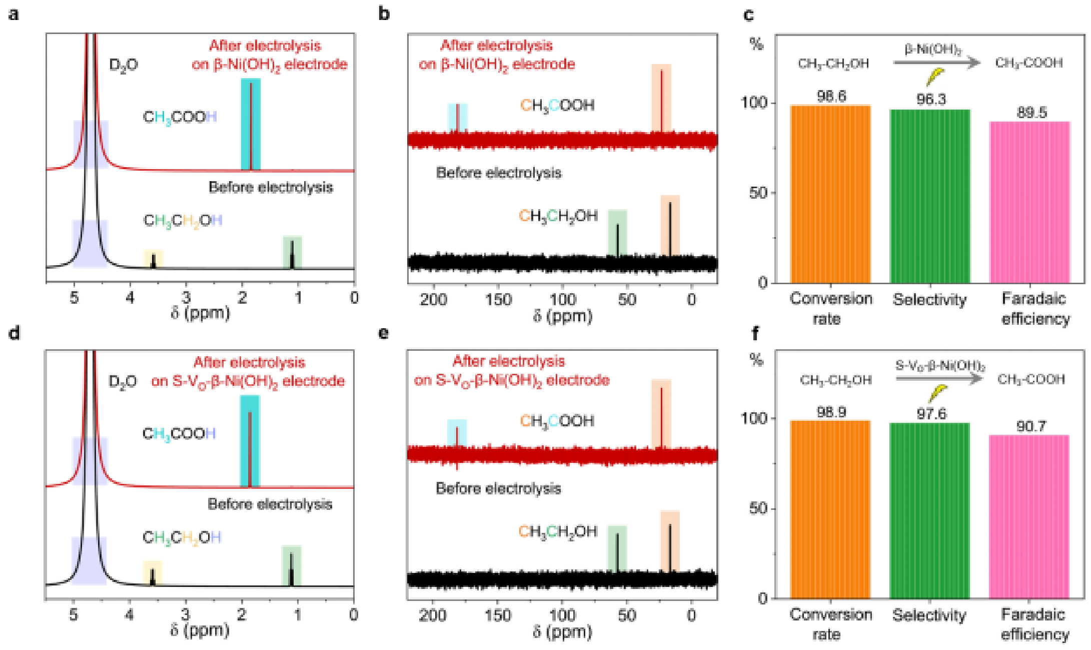

Figure S14 (a, d) ^{1}H NMR spectra of electrolytes (1 M KOH with 50 mM CH_3-CH_2OH) before/after electrolysis for PAOR on β-$Ni(OH)_2$ (a) and S-V_O-β-$Ni(OH)_2$ (d). (b, e) ^{13}C NMR spectra of electrolytes (1 M KOH with 50 mM CH_3-CH_2OH) before and after for PAOR onβ-$Ni(OH)_2$ (b) and S-V_O-β-$Ni(OH)_2$ (e). (c, f) Conversion rates, selectivities, and Faradaic efficiencies for the electrooxidations of ethanol on β-$Ni(OH)_2$ (c) and S-V_O-β-$Ni(OH)_2$ (f). Both β-$Ni(OH)_2$ and S-V_O-β-$Ni(OH)_2$ can efficiently catalyze the electrooxidation of CH_3-CH_2OH to CH_3-COOH. The conversion rate of CH_3-CH_2OH and selectivity of CH_3-COOH are close to

~100%. The Faraday efficiency is relatively low (close to ~90%) due to the low electrolysis efficiency in a low substrate concentration.

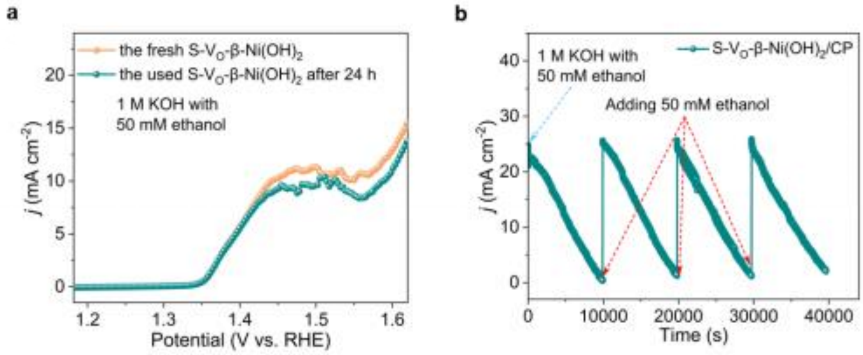

Figure S15 (a) Anodic polarization curves of the fresh S-V$_O$-β-Ni(OH)$_2$ and the used S-V$_O$-β-Ni(OH)$_2$ electrode after 24 hours of air exposure in 1 M KOH with 50 mM ethanol. (b) Long-term test (four consecutive electrolysis) of PAOR on S-V$_O$-β-Ni(OH)$_2$/CP electrode in 1 M KOH with 50 mM ethanol at the potential of 1.45 V. The fresh S-V$_O$-β-Ni(OH)$_2$ exhibited excellent PAOR performance, and the used S-V$_O$-β-Ni(OH)$_2$ electrode after 24 hours of air exposure also showed excellent PAOR performance. Besides, the PAOR performance of S-V$_O$-β-Ni(OH)$_2$ remained stable during consecutive electrolysis, indicating the stability of S-V$_O$-β-Ni(OH)$_2$ in the PAOR system.

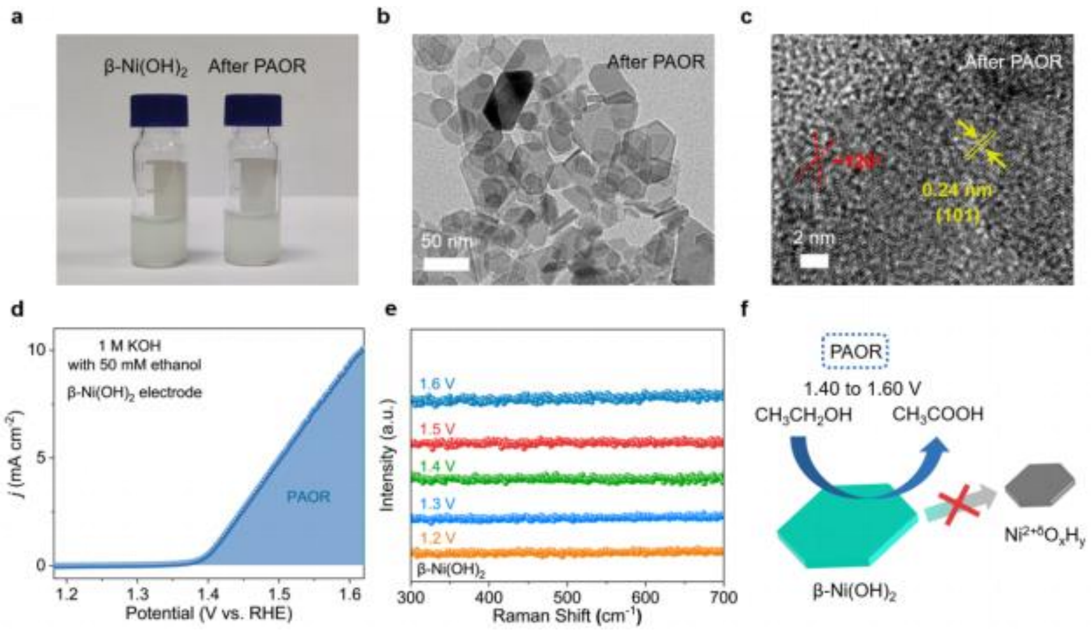

Figure S16 (a) Photograph of fresh β-Ni(OH)$_2$ and the β-Ni(OH)$_2$ after PAOR suspensions. (b, c) TEM and HR-TEM images of β-Ni(OH)$_2$ nanosheets after PAOR. (d) Anodic polarization curve of β-Ni(OH)$_2$ in the PAOR system. (e) *In situ* Raman spectra of β-Ni(OH)$_2$ in the PAOR system (1 M KOH with 0.5 M ethanol) at different potentials. (f) Schematic illustration showing the surface species evolution of the β-Ni(OH)$_2$ electrode in the PAOR system. The characterization before and after PAOR was performed to identify the catalyst function of primary alcohol electrooxidation reaction over catalysts. The potential of electrolysis and electrolysis times are 1.45 V_{RHE} and one

hour, respectively. Unlike the OER system of S-V_O-β-$Ni(OH)_2$, the color of β-$Ni(OH)_2$ after PAOR remains turquoise, suggesting that the β-$Ni(OH)_2$ electrode might maintain unchanged during PAOR (Figure S16a). According to TEM image, β-$Ni(OH)_2$ nanosheets after PAOR are still regular hexagonal nanosheets, and the lattice structure remains intact (Figure S16b and S16c). Anodic polarization curve shows that reaction current of PAOR over β-$Ni(OH)_2$ increases sharply at potentials above about 1.40 V (Figure S16d). *In situ* Raman spectra proved that $Ni^{2+\delta}$ species could not be accumulated during PAOR over β-$Ni(OH)_2$ (Figure S16e). The morphology and crystal structure of β-$Ni(OH)_2$ can remain unchanged without the formation of $Ni^{2+\delta}O_xH_y$ species during PAOR (Figure S16f).

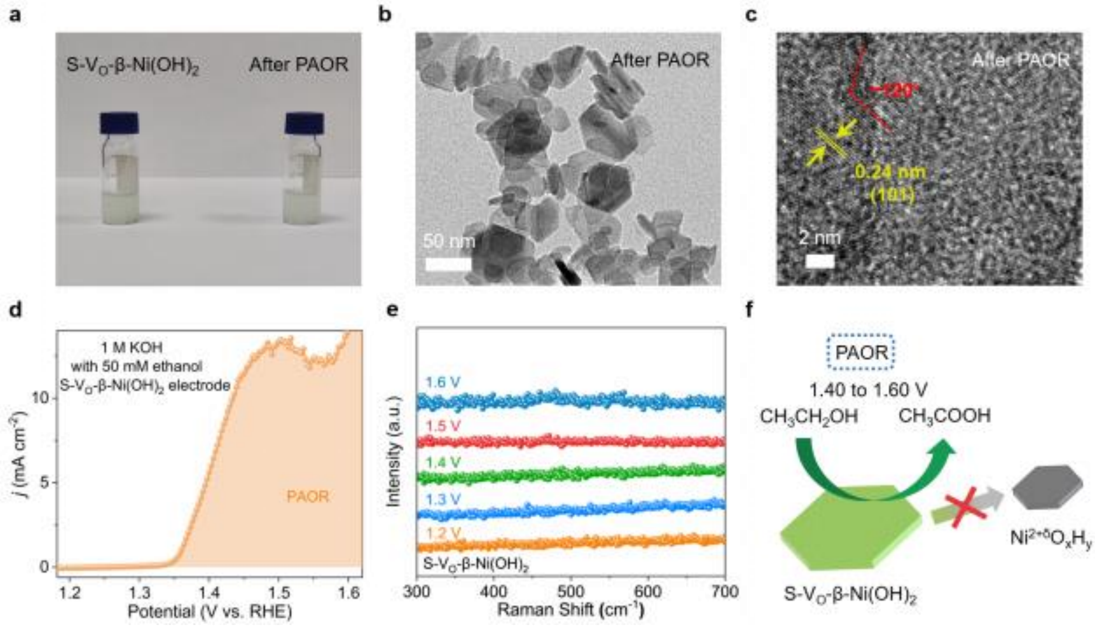

Figure S17 (a) Photograph of fresh S-V_O-β-$Ni(OH)_2$ and the S-V_O-β-$Ni(OH)_2$ after PAOR suspensions. (b, c) TEM and HR-TEM images of S-V_O-β-$Ni(OH)_2$ nanosheets after PAOR. (d) Anodic polarization curve of S-V_O-β-$Ni(OH)_2$ in the PAOR system. (e) *In situ* Raman spectra of S-V_O-β-$Ni(OH)_2$ in the PAOR system at different potentials. (f) Schematic illustration showing the surface species evolution of the S-V_O-β-$Ni(OH)_2$ electrode in the PAOR system. The PAOR system of S-V_O-β-$Ni(OH)_2$ differs vastly from the OER system. The color of the S-V_O-β-$Ni(OH)_2$ electrode after PAOR remained turquoise, suggesting that the S-V_O-β-$Ni(OH)_2$ electrode may remain unchanged during PAOR (Figure S17a). TEM images show that S-V_O-β-$Ni(OH)_2$ nanosheets after PAOR still are regular hexagonal nanosheets with a complete lattice structure (Figure S17b and S17c). These results indicate that the morphology and crystal structure of S-V_O-β-$Ni(OH)_2$ remained nearly constant during PAOR. Anodic polarization curve shows that reaction current of PAOR over S-V_O-β-$Ni(OH)_2$ increases sharply at potentials above about 1.35 V (Figure S17d). *In situ* Raman spectra proved that $Ni^{2+\delta}$ species could not be accumulated during PAOR over S-V_O-β-$Ni(OH)_2$ (Figure S17e). The generation of $Ni^{2+\delta}O_xH_y$ species cannot occur during PAOR over S-V_O-β-$Ni(OH)_2$ (Figure S17f).

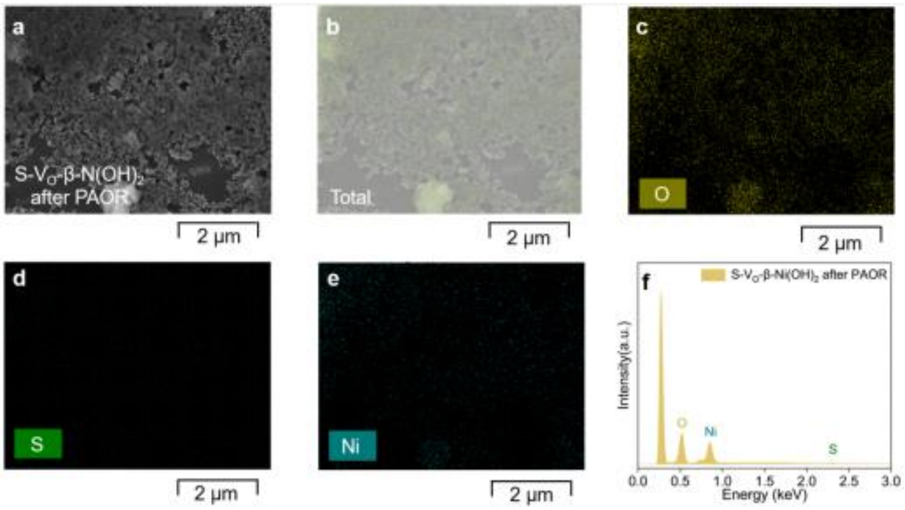

Figure S18 EDX images of the S-V_O-β-$Ni(OH)_2$ electrode after PAOR. It proves that, during PAOR over S-V_O-β-$Ni(OH)_2$, pre-electrooxidation can cause the irreversible loss of S occurs as well.

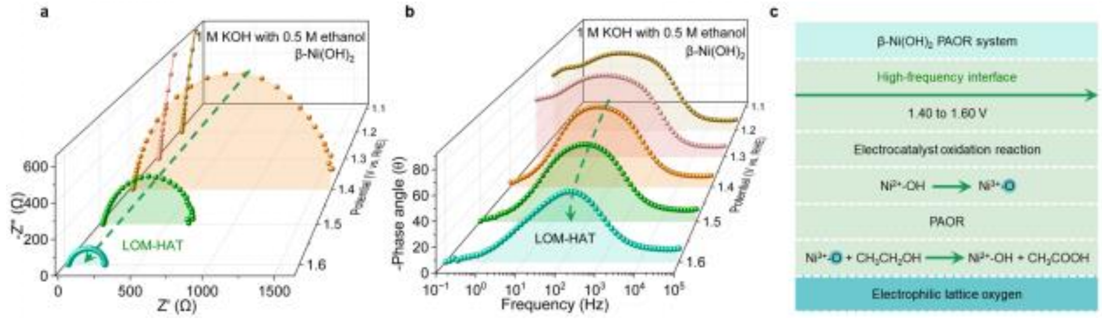

Figure S19 Nyquist plots (a), Bode plots (b), and potential-dependent behavior (c) of the β-$Ni(OH)_2$ electrode at different potentials in 1 M KOH with 0.5 M ethanol. There is only a high-frequency interface for the electrochemical behavior of β-$Ni(OH)_2$ in the electrooxidation of ethanol (the model PAOR system; electrolyte: 1 M KOH with 0.5 M ethanol) (Figure S19a). As shown in Nyquist and Bode plots of the PAOR on β-$Ni(OH)_2$, the radius of semicircle and the phase angle of the high-frequency electrochemical step decrease as the potential increases at a potential above 1.4 V (Figure S19a and S19b). It is worth noting that the low-frequency electrochemical step, *i.e.*, the electrochemical generation of electrophilic adsorbed oxygen species, does not work during the PAOR on β-$Ni(OH)_2$ (Figure S19a and S19b). These results suggest that the electrochemical step of the PAOR on β-$Ni(OH)_2$ is the high-frequency electrochemical step, *i.e.*, the electrooxidation of Ni^{2+}-OH to Ni^{3+}-O bond containing electrophilic lattice oxygen, instead of the electrochemical generation of electrophilic adsorbed oxygen species. We assume that the Ni^{3+}-O bond containing electrophilic lattice oxygen can seize the hydrogen atom of alcohols to generate carboxylic acid products and Ni^{2+}-OH bonds, thus avoiding the accumulation of Ni^{3+}-O bonds and $Ni^{2+\delta}O_xH_y$ species [12]. In brief, due to the synergy of the electrooxidation of Ni^{2+}-OH to Ni^{3+}-O and the hydrogen atom transfer (HAT) from alcohols to Ni^{3+}-O bond, PAOR takes place at the high-frequency interface, without the accumulation of Ni^{3+}-O bonds at the low-frequency interface (Figure S19c).

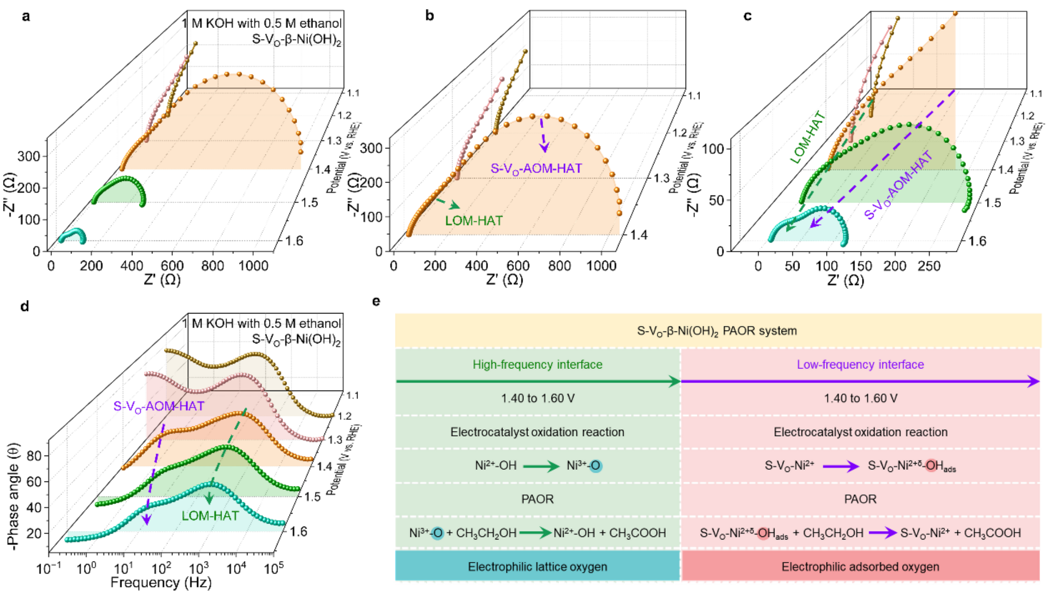

Figure S20 Nyquist plots (a-c), Bode plots (d), and potential-dependent behavior (e) of the S-V$_O$-β-Ni(OH)$_2$ electrode at different potentials in 1 M KOH with 0.5 M ethanol. For the Nyquist plots of S-V$_O$-β-Ni(OH)$_2$ in 1 M KOH with 0.5 M ethanol, there are two semicircles during the ethanol electrooxidation (the model PAOR system) at a potential above 1.4 V (Figure S20a). The radiuses of two semicircles decrease as the potential increases at a potential above 1.4 V, suggesting that both the high-frequency and low-frequency electrochemical steps play important roles in the ethanol electrooxidation (PAOR) on S-V$_O$-β-Ni(OH)$_2$ (Figure S20b and S20c). The Bode plots show an intuitive presentation of two different frequency-dependent electrochemical steps during the PAOR on S-V$_O$-β-Ni(OH)$_2$ (Figure S20d). Therefore, both the electrophilic lattice oxygen species (Ni^{3+}-O bond) and the electrophilic adsorbed oxygen species (*e.g.*, S-V$_O$-$Ni^{2+δ}$-OH$_{ads}$) function as the redox mediator to catalyze the dehydrogenation of R-CH$_2$OH to R-COOH during the PAOR on S-V$_O$-β-Ni(OH)$_2$ (Figure S20e) [12].

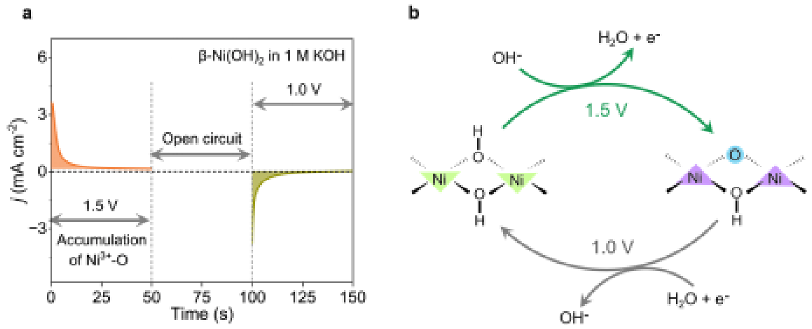

Figure S21 (a) Current density versus time of the β-Ni(OH)$_2$ electrode (0 to 50 s: 1.5 V; 50 to 100 s: open-circuit condition; 100 to 150 s: 1.0 V) in 1M KOH. (b) Corresponding schematic diagram showing the catalytic cycle including the electrooxidation of Ni^{2+}-OH to Ni^{3+}-O and the electroreduction of Ni^{3+}-O to Ni^{2+}-OH in the electrochemical testing. We carried out the multi-potential step chronoamperometric measurement to research the Ni^{2+}/Ni^{3+} redox couple for the β-Ni(OH)$_2$ electrode in 1 M KOH. The electrochemical testing of multi-potential step includes three stages (Figure S21a). 1.5 and 1.0 V were carried out at the first 50 seconds (0 to 50 s) and the third 50 seconds (100 to 150 s), respectively, and the β-Ni(OH)$_2$ electrode was under an

open-circuit condition at the second 50 seconds (50 to 100 s). At the first stage (0 to 50 s), the Ni^{3+}-O bonds containing electrophilic lattice oxygen were generated and accumulated at 1.5 V in 1 M KOH due to the electrooxidation of Ni^{2+}-OH to Ni^{3+}-O (Ni^{2+}-OH + OH^- = Ni^{3+}-O + H_2O + e^-). At the second stage (50 to 100 s), the accumulated Ni^{3+}-O bonds still existed on the electrode surface under an open circuit condition. At the third stage (100 to 150 s), the accumulated Ni^{3+}-O bonds were completely reduced to Ni^{2+}-OH bonds at 1.0 V in 1 M KOH due to the electrochemical reduction of Ni^{3+}-O to Ni^{2+}-OH bond (Ni^{3+}-O + H_2O + $e^-_{circuit}$ = Ni^{2+}-OH + OH^-). For the β-$Ni(OH)_2$ electrode in 1 M KOH, it needs a high potential (greater than 1.4 V) for the electrooxidation of Ni^{2+}-OH to Ni^{3+}-O bond, and the Ni^{3+}-O bond is relatively stable under a high potential (*e.g.*, 1.5 V) or an open circuit condition; on the other hand, the electroreduction of Ni^{3+}-O to Ni^{2+}-OH bond is spontaneous at a low potential (*e.g.*, 1.0 V) (Figure S21b).

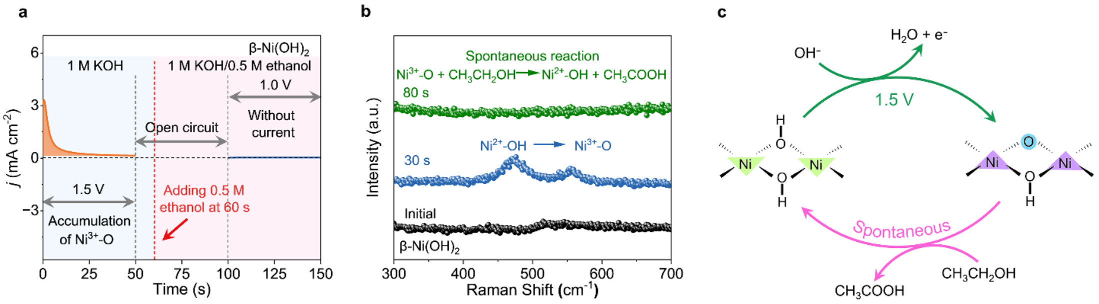

Figure S22 (a) Current density versus time of the β-$Ni(OH)_2$ electrode (0 to 50 s: 1.5 V; 50 to 100 s: open-circuit condition; 100 to 150 s: 1.0 V) in adjustable electrolytes (0 to 60 s: 1 M KOH; 60 to 150 s: 1 M KOH with 0.5 M ethanol). (b) Synchronous *in situ* Raman spectra of the β-$Ni(OH)_2$ electrode during the electrochemical testing. (c) Corresponding schematic diagram showing the catalytic cycle including the electrooxidation of Ni^{2+}-OH to Ni^{3+}-O and the spontaneous reaction between Ni^{3+}-O and R-CH_2OH. As mentioned above, the electrocatalyst function of the PAOR on β-$Ni(OH)_2$ is composed of the electrooxidation of Ni^{2+}-OH to Ni^{3+}-O and the HAT between Ni^{3+}-O and R-CH_2OH (Figure S22c). To investigate the electrocatalyst function, the HAT process should be separated from the electrooxidation of Ni^{2+}-OH to Ni^{3+}-O bond through the electrochemical testing of multi-potential step and synchronous *in situ* Raman spectra with tunable electrolytes (1 M KOH with/without 0.5 M ethanol) (Figure S22a and S22b). For the β-$Ni(OH)_2$ electrode, Ni^{3+}-O bonds were formed and accumulated at 1.50 V in 1 M KOH (0 to 50 s), and the generated Ni^{3+}-O bonds still existed in 1 M KOH under an open-circuit condition (0 to 50 s). However, after adding 0.5 M ethanol to 1 M KOH at 60 s (open-circuit conditions: 50 to 100 s), all accumulated Ni^{3+}-O bonds spontaneously reacted with ethanol to form Ni^{2+}-OH bonds, and no Ni^{3+}-O bond was identified at the third 50 seconds with the potential of 1.0 V. Hence, the electrocatalyst function of the PAOR on β-$Ni(OH)_2$, *i.e.*, LOM-HAT, includes two processes (Figure S22c): (1) the electrooxidation of Ni^{2+}-OH to Ni^{3+}-O bonds containing electrophilic lattice oxygens (Ni^{2+}-OH + OH^- = Ni^{3+}-O + H_2O + e^-), and (2) spontaneous HAT reaction between alcohols and Ni^{3+}-O bond (defined as lattice oxygen-induced HAT: LO-HAT: Ni^{3+}-O + $H_{ethanol}$ + $e^-_{circuit}$ = Ni^{2+}-OH + product).

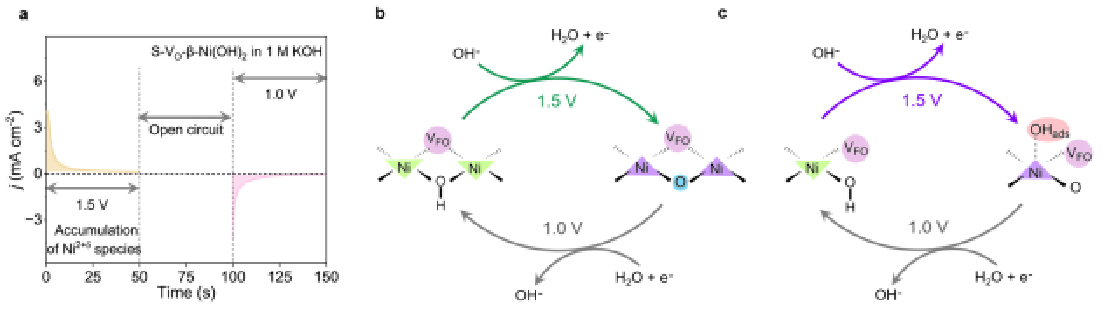

Figure S23 (a) Current density versus time of the S-V$_O$-β-Ni(OH)$_2$ electrode (0 to 50 s: 1.5 V; 50 to 100 s: open-circuit condition; 100 to 150 s: 1.0 V) in 1 M KOH. (b, c) Corresponding schematic diagrams showing the the Ni^{2+}/Ni^{3+} redox couples for S-V$_O$-β-Ni(OH)$_2$ in 1 M KOH. For the S-V$_O$-β-Ni(OH)$_2$ electrode in 1 M KOH, $Ni^{2+δ}$ species (Ni^{3+}-O bond, S-V$_O$-Ni^{3+}-O bond, and S-V$_O$-Ni^{3+}-OH$_{ads}$) were generated and accumulated at 1.50 V (0 to 50 s); the generated $Ni^{2+δ}$ species still existed under open-circuit condition (50 to 100 s) (Figure 5e); the generated $Ni^{2+δ}$ species disappeared completely at 1.0 V (100 to 150 s) because of the electroreduction of $Ni^{2+δ}$ species.

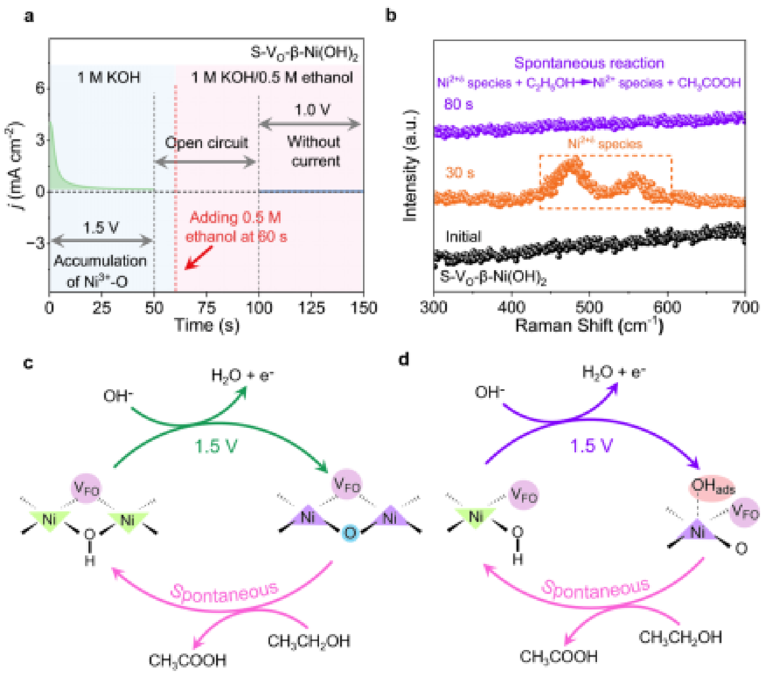

Figure S24 (a) Current density versus time of the S-V$_O$-β-Ni(OH)$_2$ electrode (0 to 50 s: 1.5 V; 50 to 100 s: open-circuit condition; 100 to 150 s: 1.0 V) in adjustable electrolytes (0 to 60 s: 1 M KOH; 60 to 150 s: 1 M KOH with 0.5 M ethanol). (b) Simultaneous *in situ* Raman spectra of S-V$_O$-β-Ni(OH)$_2$ during electrochemical testing. (c, d) Corresponding schematic diagrams showing the catalytic cycles including the electrochemical generation of S-V$_O$-Ni^{3+}-O and the spontaneous reaction between S-V$_O$-Ni^{3+}-O and R-CH$_2$OH (c), and the electrochemical generation of S-V$_O$-$Ni^{2+δ}$-OH$_{ads}$ and the spontaneous reaction between S-V$_O$-$Ni^{2+δ}$-OH$_{ads}$ and R-CH$_2$OH (d). Via combining the multi-potential step chronoamperometric measurement and *in situ* Raman spectra of S-V$_O$-β-Ni(OH)$_2$, the accumulated $Ni^{2+δ}$ species containing electrophilic lattice/adsorbed oxygen (*e.g.*, Ni^{3+}-O bond, S-V$_O$-Ni^{3+}-O bond, and S-V$_O$-$Ni^{2+δ}$-OH$_{ads}$) were

completely reduced to Ni^{2+} species while ethanol was added into the electrolyte under an open circuit condition, suggesting the spontaneous reaction between electrophilic lattice/adsorbed oxygen species and primary alcohol. Hence, in addition to Ni^{3+}-O bond, S-V_O-Ni^{3+}-O bond and S-V_O-$Ni^{2+\delta}$-OH_{ads} can spontaneously catalyze the dehydrogenation of primary alcohol as well.

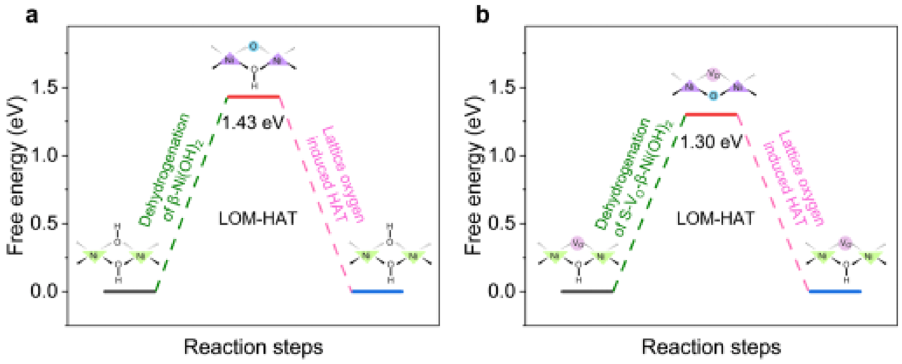

Figure S25 DFT calculations for LOM-HAT in AORs on β-$Ni(OH)_2$ (001) (a) and S-V_O-LOM-HAT in AORs on S-V_O-β-$Ni(OH)_2$ (001) (b). We have proved that LOM-HAT includes the electrochemical generation of Ni^{3+}-O (Ni^{2+}-OH + OH^- = Ni^{3+}-O + H_2O + e^-) and spontaneous lattice oxygen-induced HAT (Ni^{3+}-O + X-H + $e^-_{circuit}$ = Ni^{2+}-OH + X·) (Figure S22), and S-V_O-LOM-HAT includes the electrochemical generation of S-V_O-Ni^{3+}-O (S-V_O-Ni^{2+}-OH + OH^- = S-V_O-Ni^{3+}-O + H_2O + e^-) and spontaneous electrophilic lattice oxygen-induced HAT (S-V_O-Ni^{3+}-O + X-H + $e^-_{circuit}$ = S-V_O-Ni^{2+}-OH + X·) (Figure S24). Therefore, The electrochemical generation of Ni^{3+}-O/S-V_O-Ni^{3+}-O is the rate-limiting step in the LOM-HAT/S-V_O-LOM-HAT during AORs on β-$Ni(OH)_2$/S-V_O-β-$Ni(OH)_2$. The free energy change (ΔG = 1.30 eV) for the generation of S-V_O-Ni^{3+}-O bond is significantly lower than that (ΔG = 1.43 eV) for the generation of Ni^{3+}-O bond, indicating that the formation of S-V_O-Ni^{3+}-O bond is thermodynamically easier than the formation of Ni^{3+}-O bond.

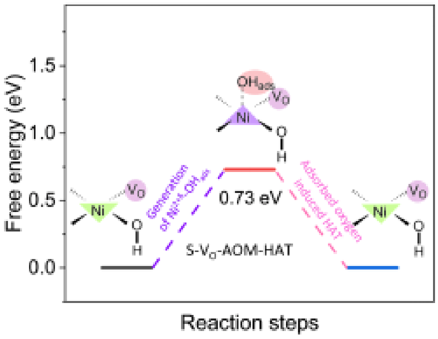

Figure S26 DFT calculation for S-V_O-AOM-HAT in AOR on S-V_O-β-$Ni(OH)_2$ (001). We have proved that S-V_O-AOM-HAT includes the electrochemical generation of S-V_O-$Ni^{2+\delta}$-OH_{ads} (S-V_O-Ni^{2+} + OH^- = S-V_O-$Ni^{2+\delta}$-OH_{ads} + e^-) and the spontaneous electrophilic adsorbed oxygen-induced HAT (S-V_O-$Ni^{2+\delta}$-OH_{ads} + X-H + $e^-_{circuit}$ = S-V_O-Ni^{2+} + H_2O + X·) (Figure S24). The generation of S-V_O-$Ni^{2+\delta}$-OH_{ads} is the rate-limiting step in the S-V_O-AOM-HAT during AORs on S-V_O-β-$Ni(OH)_2$. The formation of S-V_O-$Ni^{2+\delta}$-OH_{ads} (ΔG = 0.73 eV) is thermodynamically easier than the formation of Ni^{3+}-O bonds (ΔG = 1.43 eV).

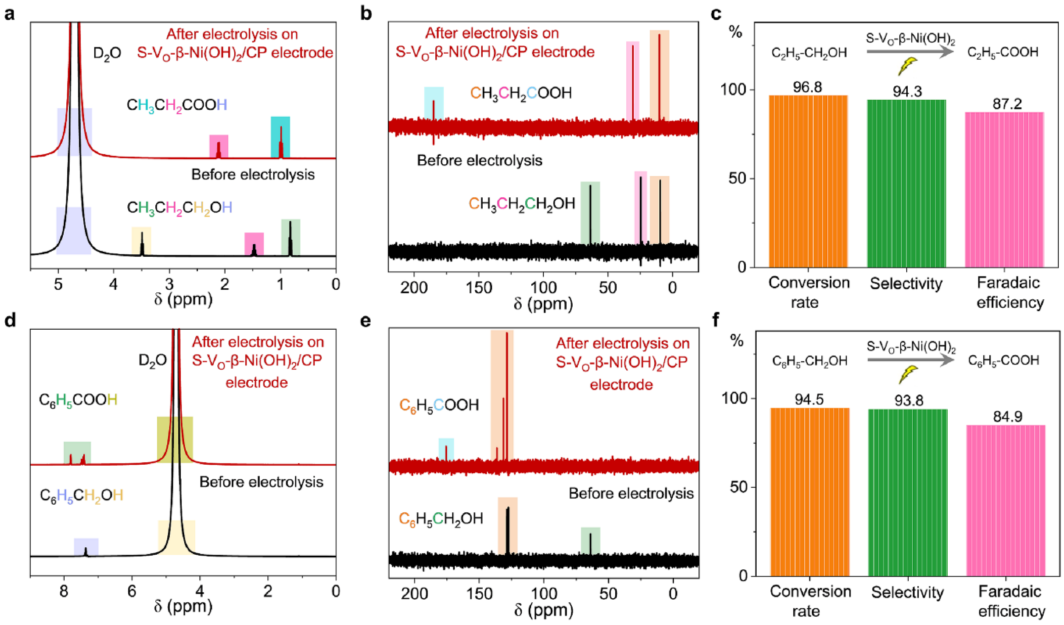

Figure S27 (a, d) ^{1}H NMR spectra for electrolytes before and after electrolysis (1 M KOH with 50 mM R-CH_2OH) for the electrooxidations of n-propyl alcohol (a) and benzyl alcohol (d). (b, e) ^{13}C NMR spectra for electrolytes before and after electrolysis for the electrooxidations of n-propyl alcohol (b) and benzyl alcohol (e). Conversion rates, selectivities, and Faradaic efficiencies for the electrooxidations of n-propyl alcohol (c) and benzyl alcohol (f) on S-V_O-β-$Ni(OH)_2$. For the S-V_O-β-$Ni(OH)_2$ electrode, the electrooxidation products of CH_3-CH_2OH, CH_3-CH_2-CH_2OH, and C_6H_5-CH_2OH are CH_3-COOH, CH_3-CH_2-COOH, and C_6H_5-COOH, respectively (Figures S14 and S27). As to different PAOR systems based on S-V_O-β-$Ni(OH)_2$, the conversion rates of primary alcohols and the selectivities of R-COOH are close to ~100%. Due to the low electrolytic efficiency for the PAOR system with a low concentration of nucleophile, Faradaic efficiencies are relatively low, yet above 84%.

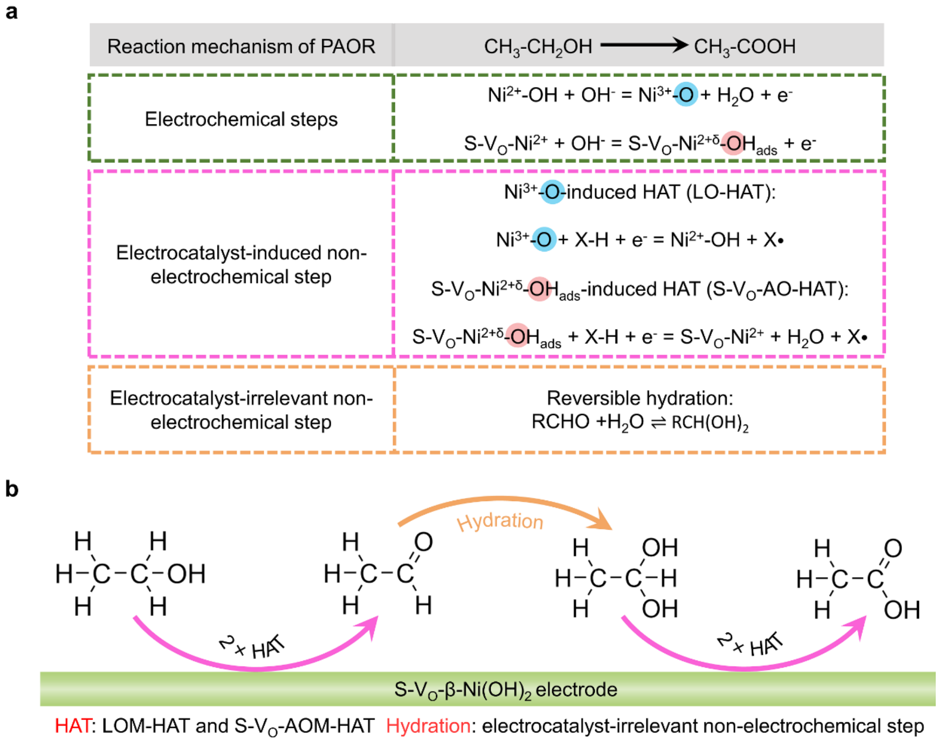

Figure S28 (a) Electrochemical and non-electrochemical steps in the PAOR on S-V_O-β-$Ni(OH)_2$. (b) Schematic diagram showing the electrooxidation of CH_3-CH_2OH on S-V_O-β-$Ni(OH)_2$. The PAOR on S-V_O-β-$Ni(OH)_2$ comprises the electrochemical step, electrocatalyst-induced non-electrochemical step, and electrocatalyst-irrelevant non-electrochemical step (Figure S28a). Electrochemical step include (1) the electrochemical generation of Ni^{3+}-O and (2) the electrochemical generation of S-V_O-$Ni^{2+\delta}$-OH_{ads}. Electrocatalyst-induced non-electrochemical steps include (1) LO-HAT and (2) AO-HAT. The electrocatalyst-irrelevant non-electrochemical step is reversible hydration reaction of R-CHO. On the S-V_O-β-$Ni(OH)_2$ electrode, CH_3-CH_2OH can be electrochemically oxidized to CH_3-COOH due to the synergistic effect between the LOM-HAT/S-V_O-AOM-HAT and hydration of CH_3-CHO (Figure S28b). In the first step, CH_3-CH_2OH loses two hydrogen atoms due to LOM-HAT and S-V_O-AOM-HAT, resulting in CH_3-CHO. Afterwards, a nucleophilic attack by water molecules on CH_3-CHO leads to the formation of aldehyde hydrates (CH_3-$CH(OH)_2$). In the end, CH_3-$CH(OH)_2$ undergoes two steps HAT processes to generate CH_3-COOH.

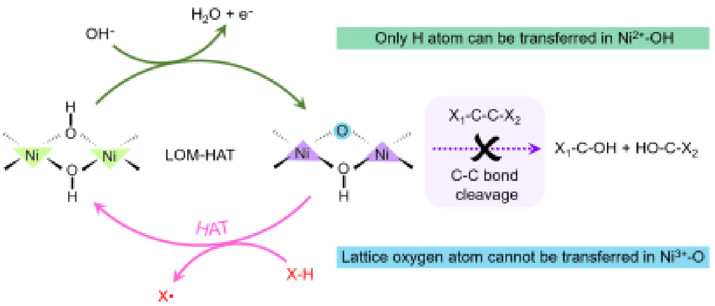

Figure S29 Schematic diagram showing lattice oxygen-mediated mechanism (LOM) for AORs on β-$Ni(OH)_2$. For AORs on β-$Ni(OH)_2$, the only redox mediator is the Ni^{3+}-O bond containing

electrophilic lattice oxygen. The electrophilic lattice oxygen in the Ni^{3+}-O bond can only serve as a hydrogen acceptor during AORs, and the electrophilic lattice oxygen cannot be transferred from Ni site to attack the C-C bond to catalyze the C-C bond cleavage [12, 13].

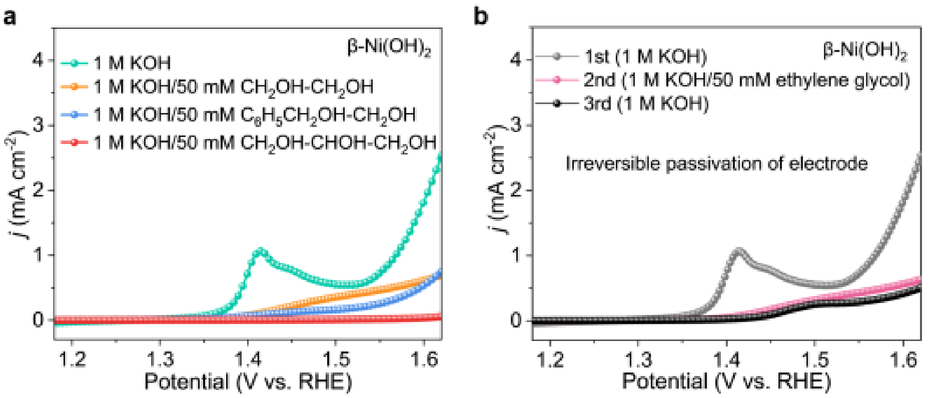

Figure S30 (a) Anodic polarization curves of β-$Ni(OH)_2$ in the POR system (in 1 M KOH with 50 mM polyols, *e.g.*, ethylene glycol, phenethyleneglycol, and glycerol). (b) Linear sweep voltammetry (LSV) curves of the β-$Ni(OH)_2$ electrode (the second LSV curve was measured in 1 M KOH with 50 mM ethylene glycol, and the others were measured in 1 M KOH). β-$Ni(OH)_2$ is unable to catalyze POR involving the cleavage of C-C bonds, leading to poor POR performances (Figure S30a). The β-$Ni(OH)_2$ electrode surface can be passivated during the ethylene glycol electrooxidation (the model POR system). Compared with the 1st LSV curve of β-$Ni(OH)_2$ in OER system, there is no Ni^{2+}/Ni^{3+} oxidation peak for the LSV curve of the passivated β-$Ni(OH)_2$ electrode in the OER system (the 3rd LSV curve), and the OER performance of the passivated β-$Ni(OH)_2$ electrode is significantly lower than that of the fresh β-$Ni(OH)_2$ electrode.

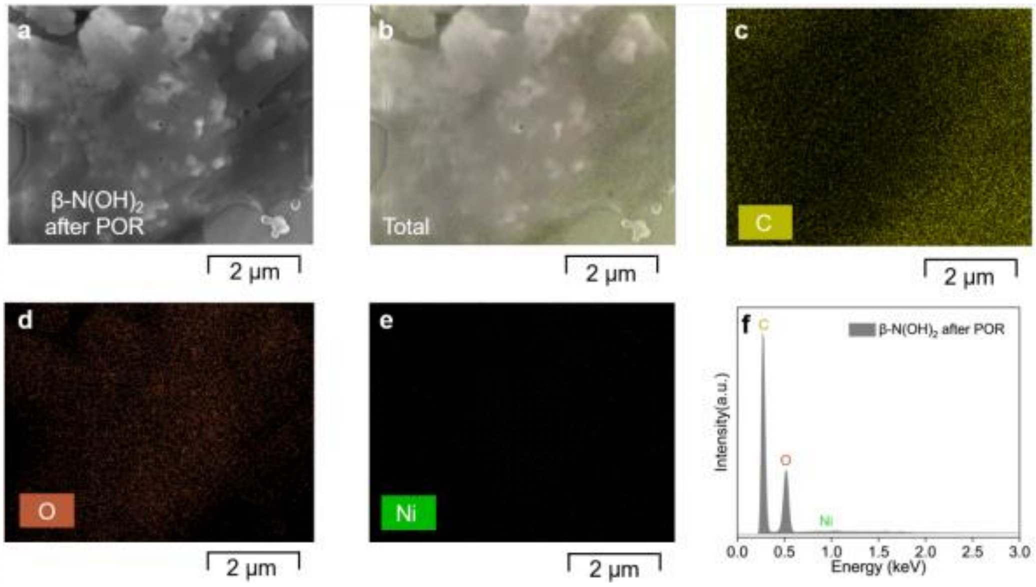

Figure S31 EDX images of the β-$Ni(OH)_2$ electrode after POR. Obviously, the β-$Ni(OH)_2$ nanosheets after POR were encapsulated by polymers. It is evident from this result that an insoluble passivation film can be formed in POR over β-$Ni(OH)_2$.

Reaction mechanism of POR on β-Ni(OH)$_2$	
Electrochemical step	Ni^{2+}-OH + OH^- = Ni^{3+}-O + H_2O + e^-
Electrocatalyst-induced non-electrochemical step	Ni^{3+}-O-induced HAT (LO-HAT): Ni^{3+}-O + R-CHOH-CH_2OH + e^- = Ni^{2+}-OH + carbonyl compounds/C-O free radical
Electrocatalyst-irrelevant non-electrochemical step	Aldol condensation and free radical coupling

Figure S32 Electrochemical and non-electrochemical steps in the POR on β-Ni(OH)$_2$. The POR on β-Ni(OH)$_2$ comprises the electrochemical steps (the electrochemical generation of Ni^{3+}-O), electrocatalyst-induced non-electrochemical steps (LO-HAT), and electrocatalyst-irrelevant non-electrochemical steps. Electrocatalyst-irrelevant non-electrochemical steps are coupling reactions between dehydrogenation intermediates, *i.e.*, (1) Free radical coupling reactions, and (2) condensation reactions.

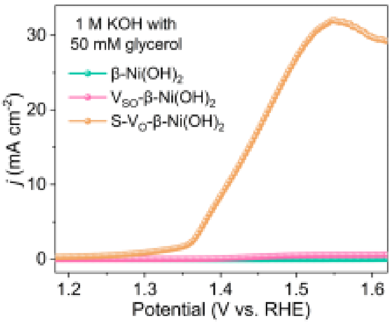

Figure S33 Anodic polarization curves of β-Ni(OH)$_2$, V_{SO}-β-Ni(OH)$_2$, and S-V_O-β-Ni(OH)$_2$ in 1 M KOH with 50 mM glycerol. Both β-Ni(OH)$_2$ and V_{SO}-β-Ni(OH)$_2$ cannot catalyze the electrooxidation of glycerol to formic acids involving the C-C bond cleavage, and both β-Ni(OH)$_2$ and V_{SO}-β-Ni(OH)$_2$ can be passivated in the POR system with glycerol substrate. S-V_O-β-Ni(OH)$_2$ exhibits an excellent performance for the electrooxidation of glycerol to formic acids, and the passivation of electrode cannot be observed during glycerol electrooxidation over S-V_O-β-Ni(OH)$_2$.

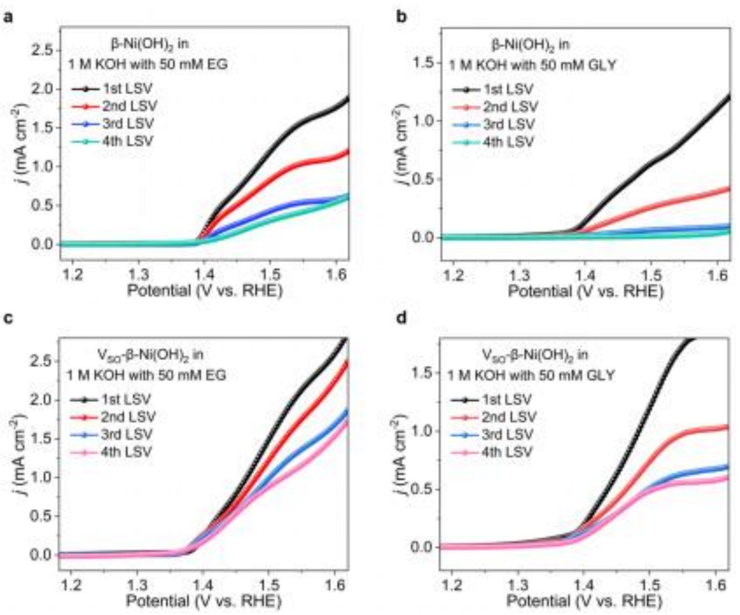

Figure S34 Anodic polarization curves of β-$Ni(OH)_2$ (a) and V_{SO}-β-$Ni(OH)_2$ (c) in 1 M KOH with 50 mM ethylene glycol. Anodic polarization curves of β-$Ni(OH)_2$ (b) and V_{SO}-β-$Ni(OH)_2$ (d) in 1 M KOH with 50 mM glycerol. The POR activities of β-$Ni(OH)_2$ and V_{SO}-β-$Ni(OH)_2$ were worsened with cycle time increasing because the passivation film continued to grow during cycling. Eventually, the passivated β-$Ni(OH)_2$ and V_{SO}-β-$Ni(OH)_2$ electrodes have almost no POR activity.

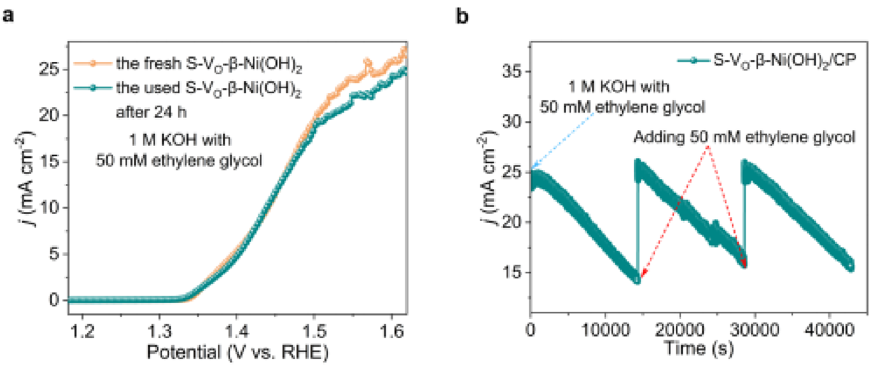

Figure S35 (a) Anodic polarization curves of the fresh S-V_O-β-$Ni(OH)_2$ and the used S-V_O-β-$Ni(OH)_2$ electrode after 24 hours of air exposure in 1 M KOH with 50 mM ethylene glycol. (b) The long-term test (three consecutive electrolysis) of POR on S-V_O-β-$Ni(OH)_2$/CP electrode in 1 M KOH with 50 mM ethylene glycol at the potential of 1.45 V. The fresh S-V_O-β-$Ni(OH)_2$ exhibited excellent POR performance, and the used S-V_O-β-$Ni(OH)_2$ electrode after 24 hours of air exposure also showed excellent EGOR performance. Besides, the EGOR performance of S-V_O-β-$Ni(OH)_2$ remained stable during consecutive electrolysis, indicating the sustained effect of oxygen vacancy-induced catalytic mechanism during EGOR on S-V_O-β-$Ni(OH)_2$.

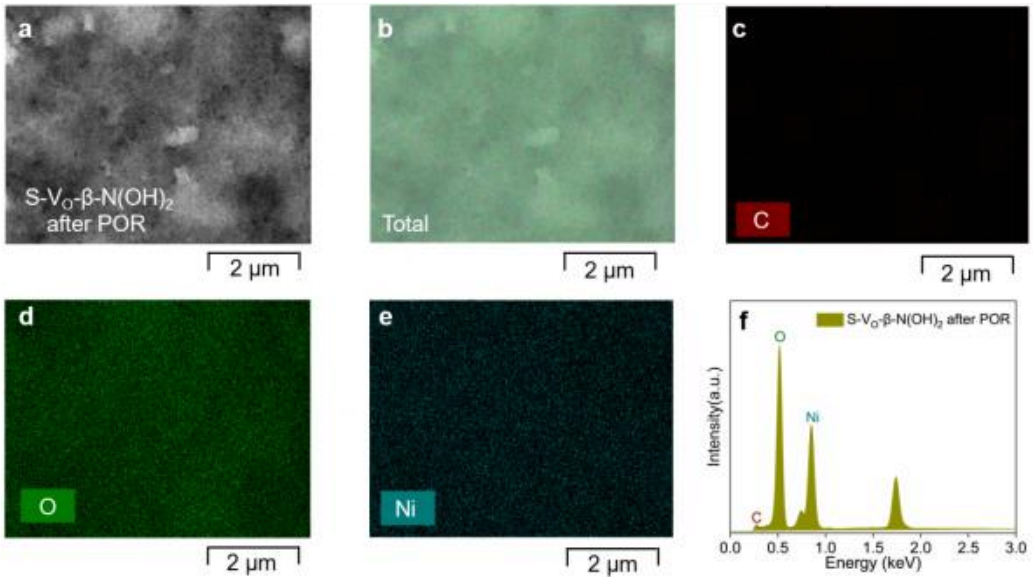

Figure S36 EDX images of the S-V_O-β-$Ni(OH)_2$ electrode after POR. As shown in the EDX images of S-V_O-β-$Ni(OH)_2$ nanosheets after POR, the passivation film containing polymers is not observed. Hence, S-V_O-β-$Ni(OH)_2$ cannot be passivated during POR.

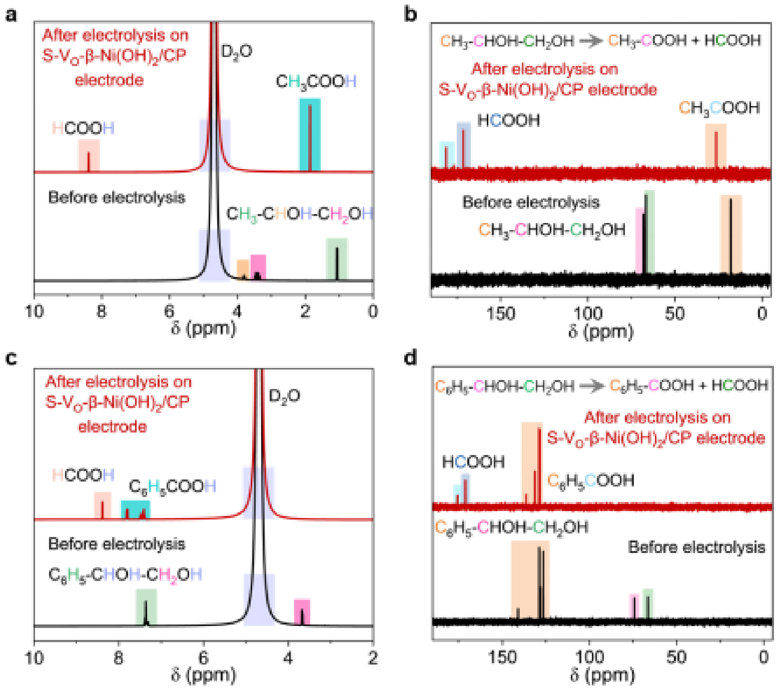

Figure S37 (a, c) ^{1}H NMR spectra for electrolytes before and after electrolysis (1 M KOH with 50 mM R-CHOH-CH_2OH) for the electrooxidations of 1,2-propylene glycol (a) and phenylethylene glycol (c) on S-V_O-β-$Ni(OH)_2$. (b, d) ^{13}C NMR spectra for electrolytes before and after electrolysis for the electrooxidations of 1,2-propylene glycol (b) and phenylethylene glycol (d) on S-V_O-β-$Ni(OH)_2$. In the POR system with S-V_O-β-$Ni(OH)_2$ electrode, one CH_3-CHOH-CH_2OH molecule can be electrochemically oxidized to one CH_3-COOH molecule and one HCOOH molecule (Figure S37a and S37b); one C_6H_5-CHOH-CH_2OH molecule can be electrochemically oxidized to one C_6H_5-COOH molecule and one HCOOH molecule (Figure S37c and S37d). In summary, on the S-V_O-β-$Ni(OH)_2$ electrode, one R-CHOH-CH_2OH molecule can be

electrochemically oxidized to one R-COOH molecule and one HCOOH molecule, accompanied by the cleavage of C-C bond.

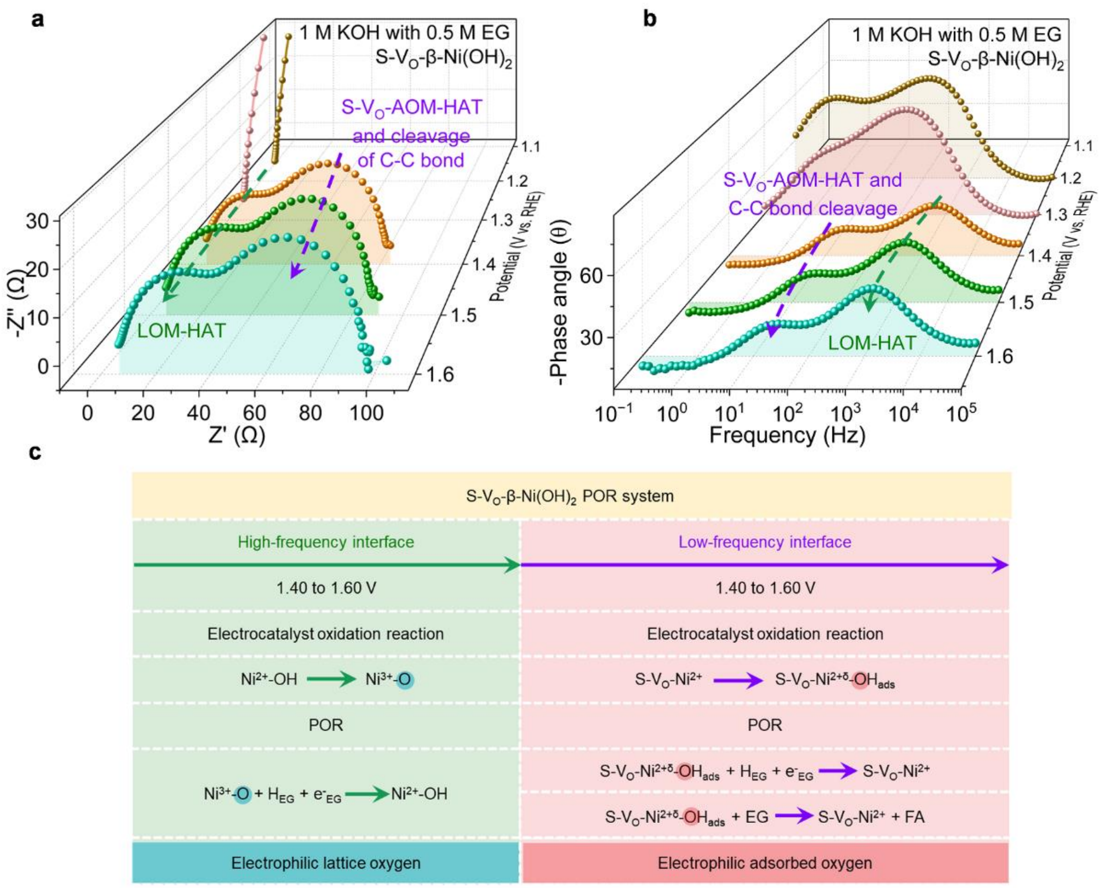

Figure S38 Nyquist plots (a), Bode plots (b), and potential-dependent behavior (c) of the S-V_O-β-$Ni(OH)_2$ electrode at different potentials in 1 M KOH with 0.5 M ethylene glycol. For the Nyquist plots of S-V_O-β-$Ni(OH)_2$ in 1 M KOH with 0.5 M ethylene glycol, there are two semicircles during the ethylene glycol electrooxidation (the model POR system), and the radiuses of two semicircles decrease as the potential increases at a potential above 1.4 V (Figure S38a). Consequently, both the electrophilic lattice oxygen species (*e.g.*, Ni^{3+}-O) and the electrophilic adsorbed oxygen species (*e.g.*, S-V_O-$Ni^{2+\delta}$-OH_{ads}) function as the redox mediator to catalyze the oxidation of R-CHOH-CH_2OH to R-COOH and HCOOH accompanied with the C-C bond cleavage during the POR on S-V_O-β-$Ni(OH)_2$ (Figure S38c).

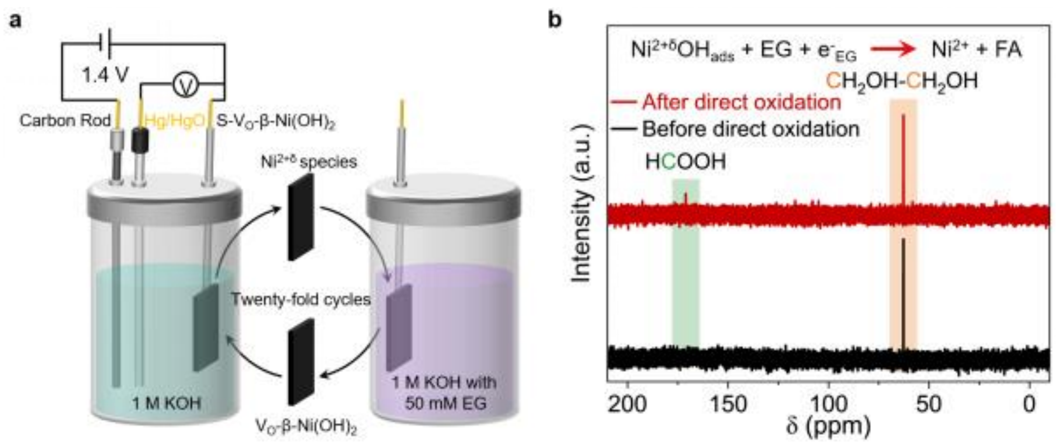

Figure S39 (a) Schematic diagram showing the verification test for the spontaneous reaction between $Ni^{2+\delta}$ species and R-CH_2OH-CH_2OH. (b) ^{13}C NMR spectra of the electrolyte (1 M KOH with 50 mM EG) before and after the twenty-fold cycles of reaction between EG and $Ni^{2+\delta}$ species. We carried out the product detection for spontaneous reactions between $Ni^{2+\delta}$ species and R-CHOH-CH_2OH. Firstly, the controlled potential electrolysis of S-V_O-β-$Ni(OH)_2$ loading on carbon paper (S-V_O-β-$Ni(OH)_2$/CP; 1 cm^2) in 1 M KOH was run at 1.5 V for 200 s to enrich $Ni^{2+\delta}$ species on the electrode. Secondly, the S-V_O-β-$Ni(OH)_2$/CP electrode containing $Ni^{2+\delta}$ species was transferred to the solution (1 M KOH with 50 mM ethylene glycol) to oxidize ethylene glycol. Thirdly, after the sufficient reaction between $Ni^{2+\delta}$ species and ethylene glycol, the electrode was rinsed with deionized water, and then transferred to the electrolyte (1 M KOH) (Figure S39a). We repeated the cycle described above twenty times to increase the concentration of reaction product in the solution. As shown in Figure S39b, a small number of formic acid was generated in the solution after the reaction between $Ni^{2+\delta}$ species and ethylene glycol. This result fully proves that $Ni^{2+\delta}$ species (*e.g.*, Ni^{3+}-O and S-V_O-$Ni^{2+\delta}$-OH_{ads}) can spontaneously catalyze the oxidative C-C bond cleavage of ethylene glycol to generate formic acid. Given that Ni^{3+}-O bond cannot be used for C-C bond cleavage, S-V_O-$Ni^{2+\delta}$-OH_{ads} is the only specie that is able to catalyze the cleavage of C-C bond spontaneously.

Reaction mechanism of POR	R-CHOH-CH_2OH ⟶ RCOOH + HCOOH
Electrochemical steps	Ni^{2+}-OH + OH^- = Ni^{3+}-O + H_2O + e^-<br>S-V_O-Ni^{2+} + OH^- = S-V_O-$Ni^{2+\delta}$-OH_{ads} + e^-
Electrocatalyst-induced non-electrochemical step	Ni^{3+}-O-induced HAT (LO-HAT):<br>Ni^{3+}-O + X-H + e^- = Ni^{2+}-OH + X•<br>S-V_O-$Ni^{2+\delta}$-OH_{ads}-induced HAT (S-V_O-AO-HAT):<br>S-V_O-$Ni^{2+\delta}$-OH_{ads} + X-H + e^- = S-V_O-Ni^{2+} + H_2O + X•<br>S-V_O-$Ni^{2+\delta}$-OH_{ads}-induced C-C bond Cleavage (S-V_O-AO-Cleavage of C-C bond):<br>S-V_O-$Ni^{2+\delta}$-OH_{ads} + R-CHOH-CH_2OH + e^- = S-V_O-Ni^{2+} + $RCH(OH)_2$ + $CH_2(OH)_2$
Electrocatalyst-irrelevant non-electrochemical step	Reversible hydration:<br>RCHO +H_2O ⇌ $RCH(OH)_2$

Figure S40 Electrochemical and non-electrochemical steps in the POR on S-V_O-β-$Ni(OH)_2$. The POR on S-V_O-β-$Ni(OH)_2$ comprises the electrochemical step, electrocatalyst-induced non-electrochemical step, and electrocatalyst-irrelevant non-electrochemical step. Electrochemical steps include (1) the electrochemical generation of Ni^{3+}-O and (2) the electrochemical generation of S-V_O-$Ni^{2+\delta}$-OH_{ads}. Electrocatalyst-induced non-electrochemical steps include (1) LO-HAT, (2) AO-HAT, and (3) AO-Cleavage of C-C bond. The electrocatalyst-irrelevant non-electrochemical step is reversible hydration reaction of R-CHO.

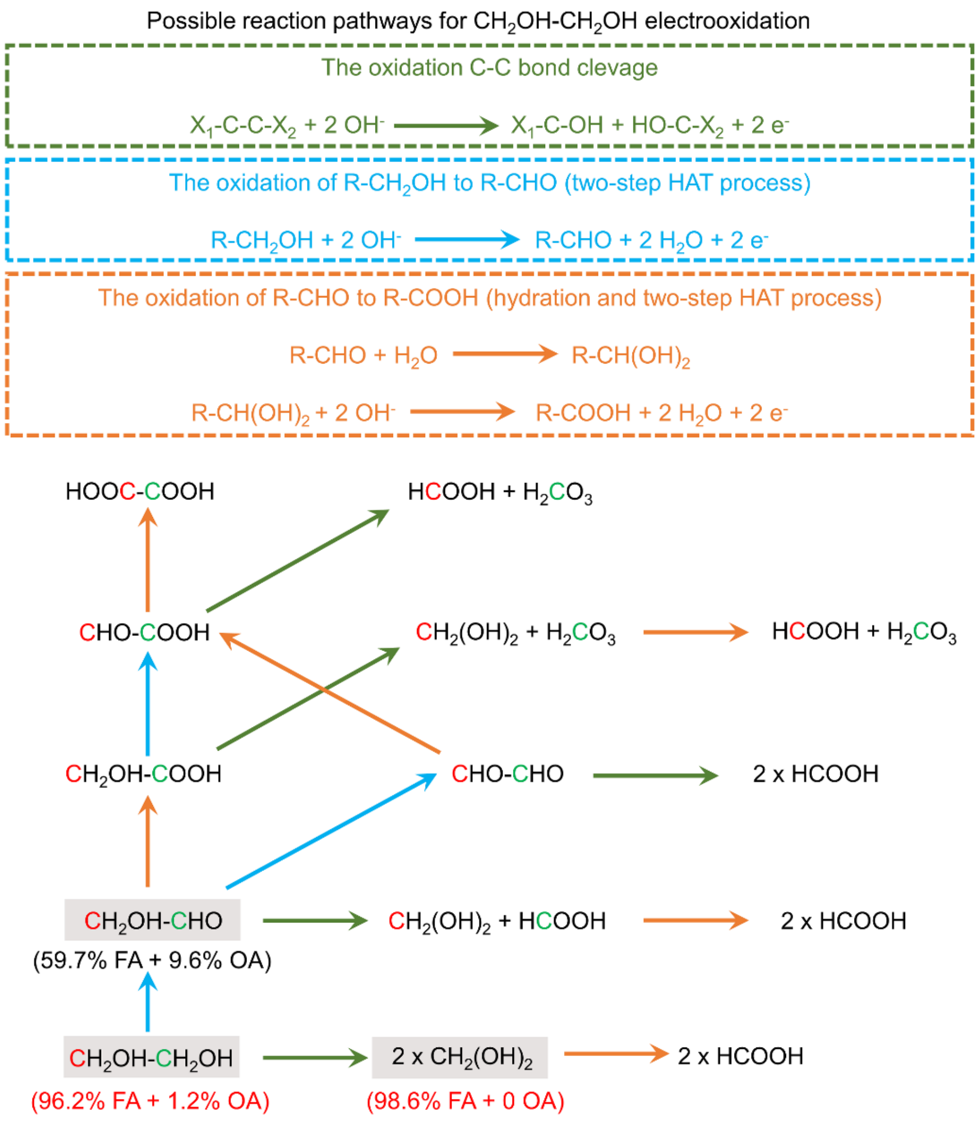

Figure S41 Schematic representation showing possible reaction pathways of the electrooxidation of CH_2OH-CH_2OH. The CH_2OH-CH_2OH electrooxidation pathway involves three basic reaction processes, *i.e.*, (1) The oxidative C-C bond cleavage of X_1-C-C-X_2 to X_1-C-OH and X_2-C-OH; (2) The electrooxidation of R-CH_2OH to R-CHO, and (3) the electrooxidation of R-CHO/R-$CH(OH)_2$ to R-COOH. For the ethylene glycol electrooxidation reaction (EGOR), there are two possible key reaction intermediates, *i.e.*, (1) formaldehyde hydrate (FALH) and (2) glycolic aldehyde (GA) [14, 15].

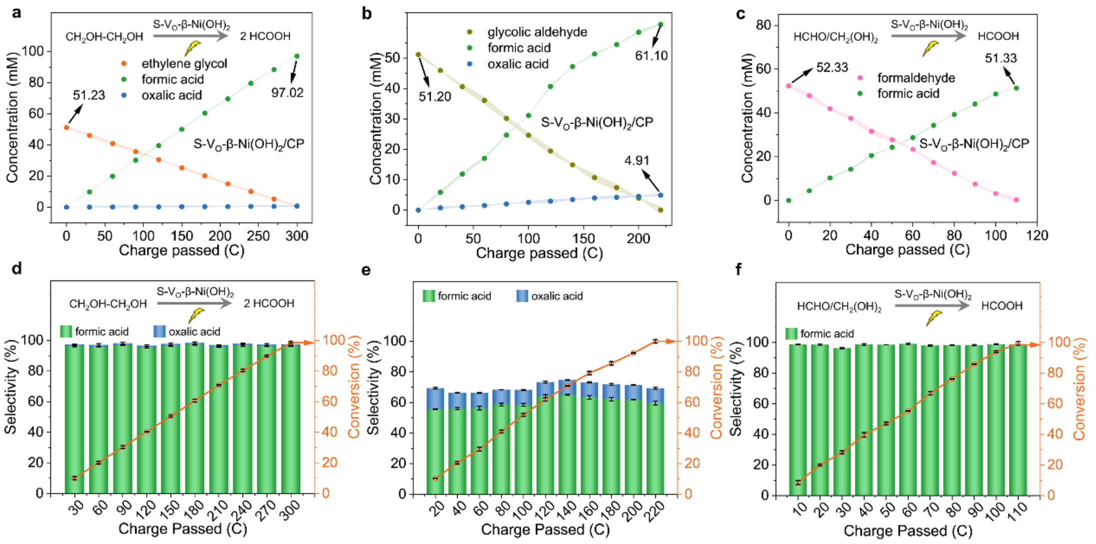

Figure S42 (a-c) Concentration changes of organic substrates and their electrooxidation products versus charge passed during electrooxidation of organic substrates over S-V_O-β-Ni(OH)$_2$ electrode, including ethylene glycol electrooxidation (a), glycolic aldehyde electrooxidation (b), and formaldehyde/formaldehyde hydrate electrooxidation (c). (d-f) Selectivities of formic acid and oxalic acid versus charge passed during electrooxidation of organic substrates over S-V_O-β-Ni(OH)$_2$ electrode, including ethylene glycol electrooxidation (d), glycolic aldehyde electrooxidation (e), and formaldehyde/formaldehyde hydrate electrooxidation (f).

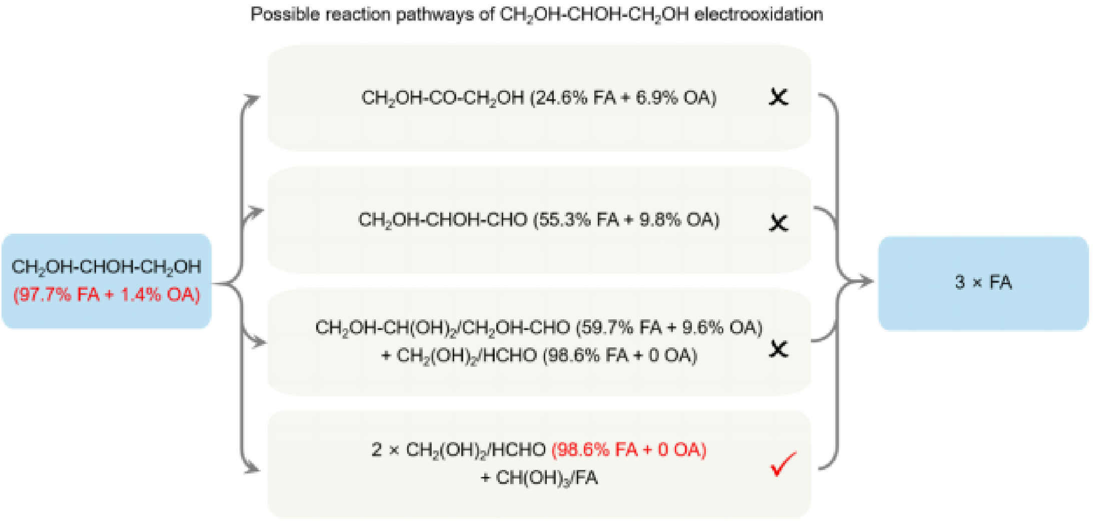

Figure S43 Schematic diagram showing possible reaction pathways of the electrooxidation of CH_2OH-CHOH-CH_2OH. In the glycerin electrooxidation reaction (GOR), the first step has four possibilities: (1) Hydroxymethyl dehydrogenation to produce glyceraldehyde (GLA), (2) Secondary hydroxyl dehydrogenation to yield dihydroxyacetone (DHA), (3) Cleavage of one C-C bond to produce glycolic aldehyde hydrate (GAH) and FALH, and (4) Cleavage of two C-C bonds to form one FA molecule and two FALH molecules [16-18].

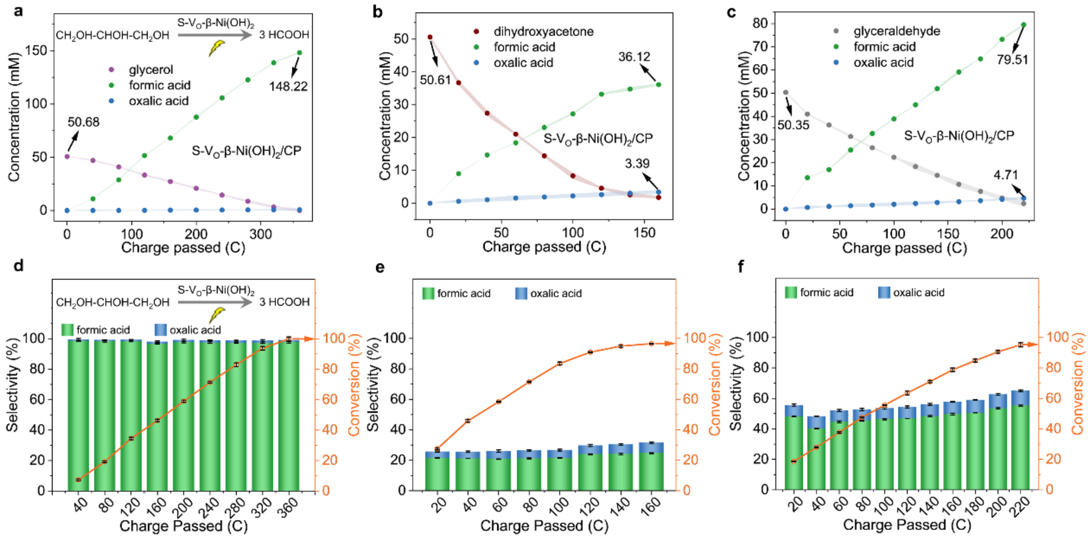

Figure S44 (a-c) Concentration changes of organic substrates and their electrooxidation products versus charge passed during electrooxidation of organic substrates over S-V_O-β-$Ni(OH)_2$ electrode, including glycerol electrooxidation (a), dihydroxyacetone electrooxidation (b), and glyceraldehyde electrooxidation (c). (d-f) Selectivities of formic acid and oxalic acid versus charge passed during electrooxidation of organic substrates over S-V_O-β-$Ni(OH)_2$ electrode, including glycerol electrooxidation (d), dihydroxyacetone electrooxidation (e), and glyceraldehyde electrooxidation (f).

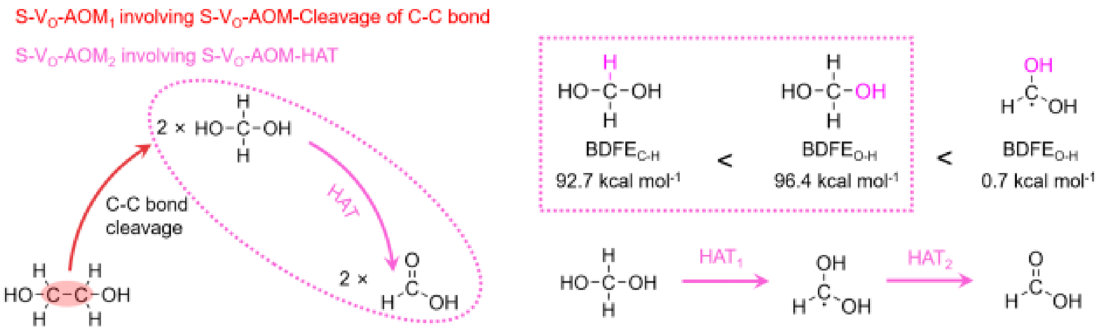

Figure S45 Reaction pathway for the electrooxidations of $H_2C(OH)_2$ to HCOOH on S-V_O-β-$Ni(OH)_2$. The S-V_O-AOM involving C-C bond cleavage preferentially occurs during the CH_2OH-CH_2OH electrooxidation on S-V_O-β-$Ni(OH)_2$, and the key reaction intermediate is $H_2C(OH)_2$. For $H_2C(OH)_2$, BDFEs of the C-H bond and the O-H bond are 92.7 and 96.4 kcal mol^{-1}, respectively. Hence, for the oxidation of $H_2C(OH)_2$ to HCOOH, the first HAT step (HAT_1) is the dehydrogenation of $H_2C(OH)_2$ to H·$C(OH)_2$, and the second step (HAT_2) is the dehydrogenation of H·$C(OH)_2$ to HCOOH.

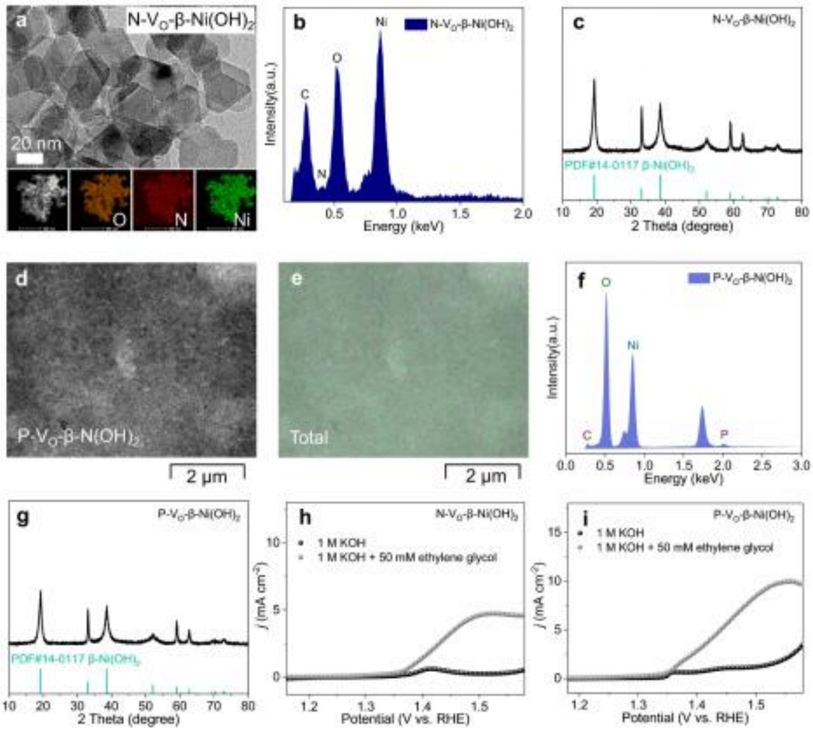

Figure S46 TEM and EDX images (a), EDX spectrum (b), and XRD pattern (c) of N-V_O-β-$Ni(OH)_2$ nanosheet. (d-g) EDX images (d, e), EDX spectrum (f), and XRD pattern (g) of P-V_O-β-$Ni(OH)_2$. Anodic polarization curves of N-V_O-β-$Ni(OH)_2$ (h) and P-V_O-β-$Ni(OH)_2$ (i) in the OER and POR systems (1 M KOH with/without ethylene glycol). Because LOM-HAT, S-V_O-AOM-HAT, and S-V_O-AOM-Cleavage of C-C bond act synergistically, S-V_O-β-$Ni(OH)_2$ exhibits superior POR performance for C-C bond cleavage of polyols into formic acids. Building on this inspiration, we incorporated different heteroatoms, such as nitrogen (N) and phosphorus (P), to fill oxygen vacancies and synthesized oxygen vacancy-filling with other heteroatoms β-$Ni(OH)_2$, *e.g.*, N-V_O-β-$Ni(OH)_2$ and P-V_O-β-$Ni(OH)_2$. The crystal structure and morphology of β-$Ni(OH)_2$, N-V_O-β-$Ni(OH)_2$, and P-V_O-β-$Ni(OH)_2$ nanosheets closely resemble each other, implying the microstructure of these samples remained mostly unaltered during the fabrication of N-V_O-β-$Ni(OH)_2$ and P-V_O-β-$Ni(OH)_2$ (Figure S46a-S46g). Both N-V_O-β-$Ni(OH)_2$ and P-V_O-β-$Ni(OH)_2$ demonstrate outstanding POR performance without experiencing electrode passivation (Figure S46h and S46i).

Supplementary Tables.

Table S1. Elemental quantification obtained by EDX spectrum analysis of β-$Ni(OH)_2$.

Element	Weight %	Atomic %	Error %	Net Int.	K Ratio	Z	A	F
C K	2.90	7.15	10.53	94.23	0.0085	1.2147	0.2425	1.0000
O K	32.54	60.26	6.04	3419.43	0.2035	1.1617	0.5383	1.0000
Ni K	64.57	32.59	3.08	1214.26	0.5796	0.8898	1.0044	1.0043

Table S2. Elemental quantification obtained by EDX spectrum analysis of S-V_O-β-$Ni(OH)_2$.

Element	Weight %	Atomic %	Error %	Net Int.	K Ratio	Z	A	F
C K	3.47	8.18	11.21	98.35	0.0090	1.2022	0.2162	1.0000
O K	33.87	59.84	6.46	3257.65	0.1965	1.1495	0.5046	1.0000
S K	4.54	4.00	4.48	461.76	0.0377	1.0209	0.8092	1.0056
Ni K	58.12	27.98	3.28	1065.90	0.5156	0.8782	1.0042	1.0059

Table S3. Ni K-edge EXAFS fitting results of β-$Ni(OH)_2$, V_{SO}-β-$Ni(OH)_2$, and S-V_O-β-$Ni(OH)_2$.

Sample	Shell	CN	R (Å)	σ^2 (Å^2)	$\triangle E_0$ (eV)	R factor
β-$Ni(OH)_2$	Ni-O	6.37	2.06	0.00814	-4.274	0.025
	Ni-Ni	5.65	3.11	0.00660		
V_{SO}-β-$Ni(OH)_2$	Ni-O	4.92	2.05	0.00767	-4.309	0.022
	Ni-Ni	4.22	3.11	0.00568		
S-V_O-β-$Ni(OH)_2$	Ni-O/S	6.15	2.06	0.01160	-4.234	0.030
	Ni-Ni	4.08	3.09	0.00823		

Table S4. Elemental quantification obtained by EDX spectrum analysis of S-V_O-β-$Ni(OH)_2$ after pre-oxidation.

Element	Weight %	Atomic %	Error %	Net Int.	K Ratio	Z	A	F
S K	0.24	0.25	29.50	16.24	0.0020	1.0512	0.7804	1.0059
Ni K	70.34	39.25	3.27	892.65	0.6431	0.9081	1.0035	1.0034
O K	29.42	60.40	6.03	2127.04	0.1913	1.1816	0.5503	1.0000

Table S5. Elemental quantification obtained by EDX spectrum analysis of S-V_O-β-$Ni(OH)_2$ after PAOR.

Element	Weight %	Atomic %	Error %	Net Int.	K Ratio	Z	A	F
S K	1.87	1.69	21.59	23.96	0.0155	1.0246	0.8016	1.0059
Ni K	60.08	29.58	4.84	139.75	0.5348	0.8817	1.0043	1.0054
O K	38.05	68.73	6.69	523.75	0.2502	1.1534	0.5701	1.0000

Table S6. Elemental quantification obtained by EDX spectrum analysis of β-$Ni(OH)_2$ after POR.

Element	Weight %	Atomic %	Error %	Net Int.	K Ratio	Z	A	F
C K	57.10	64.30	4.19	2770.57	0.4614	1.0268	0.7872	1.0000
O K	41.96	35.48	8.13	1255.96	0.1728	0.9687	0.4252	1.0000
Ni K	0.94	0.22	7.92	15.62	0.0055	0.7290	0.8047	1.0000

Table S7. Elemental quantification obtained by EDX spectrum analysis of S-V_O-β-$Ni(OH)_2$ after POR.

Element	Weight %	Atomic %	Error %	Net Int.	K Ratio	Z	A	F
C K	3.60	8.53	10.01	118.00	0.0108	1.2057	0.2484	1.0000
O K	34.63	61.55	6.00	3589.62	0.2155	1.1530	0.5397	1.0000
Ni K	61.77	29.92	3.06	1141.64	0.5499	0.8818	1.0046	1.0049

Table S8. Elemental quantification obtained by EDX spectrum analysis of P-V_O-β-$Ni(OH)_2$.

Element	Weight %	Atomic %	Error %	Net Int.	K Ratio	Z	A	F
C K	3.41	8.37	10.98	106.88	0.0098	1.2134	0.2361	1.0000
O K	31.88	58.65	6.24	3233.92	0.1944	1.1604	0.5256	1.0000
P K	1.20	1.14	7.95	113.49	0.0086	1.0117	0.7106	1.0038
Ni K	63.51	31.84	3.24	1180.36	0.5693	0.8885	1.0042	1.0046

References

1. Kresse G, Hafner J. Ab initio molecular dynamics for open-shell transition metals. *Phys Rev B*. 1993; **48**: 13115-13118.
2. Blöchl PE. Projector augmented-wave method. *Phys Rev B*. 1994; **50**: 17953-17979.
3. Kresse G, Joubert D. From ultrasoft pseudopotentials to the projector augmented-wave method. *Phys Rev B*. 1999; **59**: 1758-1775.
4. Grimme S, Antony J, Ehrlich S *et al.* A consistent and accurate ab initio parametrization of density functional dispersion correction (DFT-d) for the 94 elements H-Pu. *J Chem Phys*. 2010; **132**: 154104.
5. Qiu Z, Tai C, Niklasson GA *et al.* Direct observation of active catalyst surface phases and the effect of dynamic self-optimization in NiFe-layered double hydroxides for alkaline water splitting. *Energy Environ Sci*. 2019; **12**: 572-581.
6. Zhang J, Liu J, Xi L *et al.* Single-atom Au/NiFe layered double hydroxide electrocatalyst: Probing the origin of activity for oxygen evolution reaction. *J Am Chem Soc*. 2018; **140**: 3876-3879.
7. Faid AY, Barnett AO, Seland F *et al.* Ni/NiO nanosheets for alkaline hydrogen evolution reaction: In situ electrochemical-Raman study. *Electrochim Acta*. 2020; **361**: e137040.
8. Wang H, Casalongue HS, Liang Y *et al.* $Ni(OH)_2$ nanoplates grown on graphene as Advanced electrochemical pseudocapacitor materials. *J Am Chem Soc*. 2010; **132**: 7472-7477.
9. Xiao Z, Huang Y-C, Dong C-L *et al.* Operando identification of the dynamic behavior of oxygen vacancy-rich Co_3O_4 for oxygen evolution reaction. *J Am Chem Soc*. 2020; **142**: 12087-12095.
10. Kang J, Qiu X, Hu Q *et al.* Valence oscillation and dynamic active sites in monolayer nico hydroxides for water oxidation. *Nat Catal*. 2021; **4**: 1050-1058.

11. Xiao Z, Wang Y, Huang Y-C *et al.* Filling the oxygen vacancies in Co_3O_4 with phosphorus: An ultra-efficient electrocatalyst for overall water splitting. *Energy Environ Sci*. 2017; **10**: 2563-2569.
12. Chen W, Xie C, Wang Y *et al.* Activity origins and design principles of nickel-based catalysts for nucleophile electrooxidation. *Chem*. 2020; **6**: 2974-2993.
13. Chen W, Shi J, Xie C *et al.* Unraveling the electrophilic oxygen-mediated mechanism for alcohol electrooxidation on NiO. *Natl Sci Rev*. 2023; **10**: nwad099.
14. Qin Y, Zhang W, Wang F *et al.* Extraordinary p-d hybridization interaction in heterostructural Pd-PdSe nanosheets boosts C-C bond cleavage of ethylene glycol electrooxidation. *Angew Chem Int Ed*. 2022; **61**: e202200899.
15. Li J, Li L, Ma X *et al.* Selective ethylene glycol oxidation to formate on nickel selenide with simultaneous evolution of hydrogen. *Adv Sci*. 2023; **10**: e2300841.
16. Zhang N, Wang J, Zhang W *et al.* Self-supported PdNi dendrite on Ni foam for improving monohydric alcohol and polyhydric alcohols electrooxidation. *Fuel*. 2022; **326**: e125083.
17. Sheng H, Janes AN, Ross RD *et al.* Linear paired electrochemical valorization of glycerol enabled by the electro-Fenton process using a stable $NiSe_2$ cathode. *Nat Catal*. 2022; **5**: 716-725.
18. Mou H, Chang Q, Xie Z *et al.* Enhancing glycerol electrooxidation from synergistic interactions of platinum and transition metal carbides. *Appl Catal B*. 2022; **316**: e121648.
